# Genetic Susceptibility to Gestational Diabetes Mellitus in a Chinese Population

**DOI:** 10.3389/fendo.2020.00247

**Published:** 2020-04-22

**Authors:** Minkai Cao, Le Zhang, Ting Chen, Aiwu Shi, Kaipeng Xie, Zhengying Li, Jianjuan Xu, Zhong Chen, Chenbo Ji, Juan Wen

**Affiliations:** ^1^Department of Obstetrics, The Affiliated Wuxi Maternity and Child Health Care Hospital of Nanjing Medical University, Wuxi, China; ^2^Department of Neonatology, The Affiliated Wuxi Children's Hospital of Nanjing Medical University, Wuxi, China; ^3^Nanjing Maternity and Child Health Care Institute, Nanjing Maternity and Child Health Care Hospital, Women's Hospital of Nanjing Medical University, Nanjing, China; ^4^Department of MICU, Nanjing Maternity and Child Health Care Hospital, Women's Hospital of Nanjing Medical University, Nanjing, China

**Keywords:** polymorphism, gestational diabetes mellitus, susceptibility, *FTO*, *KCNQ1*, *MC4R*, *PROX1*

## Abstract

**Introduction:** New genetic variants associated with susceptibility to obesity and metabolic diseases have been discovered in recent genome-wide association (GWA) studies. The aim of this study was to investigate the association of theses risk variants with gestational diabetes mellitus (GDM).

**Methods:** We performed a case-control study including 964 unrelated pregnant women with GDM and 1,021 pregnant women with normal glucose tolerance (as controls). A total of 33 genetic variants confirmed by GWA studies for obesity and metabolic diseases were selected and measured.

**Results:** We observed that *FTO* rs1121980 and *KCNQ1* rs163182 conferred a decreased GDM risk in the dominant and additive model [additive model: OR (95% CI) = 0.79 (0.67–0.94), *P* = 0.007 for rs1121980; OR(95%CI) = 0.84 (0.73–0.96), *P* = 0.009 for rs163182], whereas *MC4R* rs12970134 and *PROX1* rs340841 conferred an increased GDM risk in the dominant, recessive, and additive model [additive model: OR(95%CI) = 1.25 (1.07–1.46), *P* = 0.006 for rs12970134; OR(95%CI) = 1.22 (1.07–1.39), *P* = 0.002 for rs340841). With the increasing number of risk alleles of the four significant SNPs, GDM risk was significantly increased in a dose-dependent manner (*P*_trend_ < 0.001). And the significant positive associations between the weighted genetic risk score and risk of GDM persisted. Further function annotation indicated that these four SNPs may fall on the functional elements of human pancreatic islets. The genotype-phenotype associations indicated that these SNPs may contribute to GDM by affecting the expression levels of their nearby or distant genes.

**Conclusion:** Our study suggests that *FTO* rs1121980, *KCNQ1* rs163182, *MC4R* rs12970134, and *PROX1* rs340841 may be markers for susceptibility to GDM in a Chinese population.

## Introduction

Gestational diabetes mellitus (GDM) is a major public health problem, affecting about 5–10% of all pregnancies (1). Globally the prevalence of GDM has increased dramatically in the past three decades, following worldwide trends of increasing obesity and type 2 diabetes (T2D) (2). GDM has important clinical implications because of its associated adverse neonatal outcomes (3), and its increased risk for long-term complications, including obesity and impaired glucose metabolism, in both the mother and infant (4). Additionally, women with high body mass index (BMI) and a family history of diabetes may be predisposed to an increased risk of GDM (5). Given the connection, it is plausible to hypothesize that GDM may share the common genetic susceptibilities with T2D and obesity.

Accumulating evidences suggest that genetic factors play a role in GDM (6, 7). The major genetic studies of GDM were candidate gene studies, which have successfully identified the association of risk variants for T2D with GDM, thereby confirming the genetic similarity between GDM and T2D (8, 9). Besides, it is widely acknowledged that the *FTO* (fat-mass and obesity-associated gene) is related to BMI and obesity (10). Several studies have revealed that some genetic variants in *FTO* gene could contribute to the risk of GDM (11). However, the association with GDM was not observed in all the T2D and obesity associated loci, and these associations varied by race (12). Genome-wide association (GWA) studies have so far identified a large number of single nucleotide polymorphisms (SNPs) in different genes associated with susceptibility to obesity and metabolic diseases, including genetic variants in *BDNF, FTO, GCK, GCKR, KCNQ1, MC4R, PROX1, UBE2E2*, and so on (13–16). In this study, we speculated that some of these SNPs may also influence the development of GDM. To verify this assumption and systematically evaluate the genetic similarity, we selected 33 SNPs in multiple genes and designed a case-control study of 964 GDM cases and 1,021 controls to assess the associations of the SNPs with GDM risk. Further functional annotation of the significant SNPs was also conducted.

## Materials and Methods

### Study Subjects and Design

This study was approved by the ethics committee of Women's Hospital of Nanjing Medical University (NFY201608), and all methods were carried out according to the related guidelines. The enrolment of the subjects has been described in our previous paper (17).

In brief, this case-control study was performed based on a study population of about 80,000 women who participating gestational complications screening between 2012 and 2015 in Women's Hospital of Nanjing Medical University. Using completely randomized digital table, the GDM cases and controls were randomly selected from the screening population. For all participants between 24 and 28 weeks of gestation, a glucose challenge test (GCT) was conducted. The GDM cases were defined as pregnant woman with fasting blood glucose ≥5.5 mmol/L or 2 h plasma glucose ≥8.0 mmol/L following a 75 g oral glucose tolerance test (OGTT) (18). Participants diagnosed with metabolic syndrome and related diseases before pregnancy were excluded from this study. The pregnant women without diabetes were included as controls. The controls were matched to GDM cases for age and pre-pregnancy body mass index (BMI). At last, 964 GDM patients and 1,021 controls agreed to participate in the study. All participants were unrelated ethnic Han Chinese. After written informed consent was obtained, each participant was arranged an interview and used structured questionnaires to collect demographic information and potential risk factors, such as age, parity, pre-pregnancy height and weight, family history of diabetes, and abnormal pregnancy history.

### SNPs Selection and Genotyping

Based on the data from GWAS Catalog (http://www.ebi.ac.uk/gwas/), SNPs that reach a genome-wide significant association with obesity or metabolic diseases were included for SNP selection. The traits of obesity and metabolic diseases included BMI, obesity, T2D, metabolic syndrome, fasting glucose-related traits, 2 h glucose challenge, and so on. The SNPs in hotspot susceptibility gene reported in multiple GWAS analysis with validation or in GWAS meta-analysis were given greater priority. Then, SNPs with minor allele frequency (MAF) ≥ 0.05 in Han Chinese were selected. If several SNPs were in high linkage disequilibrium (*r*^2^ > 0.8), only one SNP was genotyped. As a result, 33 SNPs reported from GWA study were selected for genotyping (Supplementary Table 1). The flow chart of SNP selection is shown in Supplementary Figure 1.

Genomic DNA was extracted from leukocyte microspheres by traditional proteinase K digestion, followed by phenol-chloroform extraction and ethanol precipitation. All SNPs were genotyped using Sequenom MassARRAY iPLEX platform (Sequenom Inc., CA). The information regarding the primers is shown in Supplementary Table 2. Genotyping was conducted blindly without knowing the subjects' case or control status. Two negative controls were used for quality control in each 384-well-plate, and more than 10% samples were randomly selected for repeated sampling, with a concordance rate of 100%. The genotyping success rates of these SNPs were all above 95.0%.

### *In silico* Analysis

In order to further understand the function of the significant SNPs in the pathogenesis of GDM, the ENCODE database (http://genome.ucsc.edu/) and the Roadmap Epigenomics database (http://genomebrowser.wustl.edu/) were used to explore whether the SNPs were located in the functional elements. In addition, PhenoScanner database (http://www.phenoscanner.medschl.cam.ac.uk/) was used to investigate the genotype-phenotypic associations of significantly correlated SNPs and their associated high-LD SNPs (*r*^2^ > 0.8 from the 1000 Genomes Project).

### Statistical Analyses

The Student's *t*-test and χ^2^-test were used to detect differences of selected characteristics and genotype frequencies of the SNPs between the GDM cases and controls for continuous variables and categorical variables, respectively. Logistic regression analyses were used to calculate the odds ratios (OR) and their 95% confidence intervals (CIs) to estimate the relationship between genotypes and GDM risk. The crude ORs were calculated by univariate logistic regression, while the adjusted ORs were calculated by multivariate logistic regression with the adjustment for age, parity, pre-pregnancy BMI, family history of diabetes, and abnormal pregnancy history. Logistic regression analyses with different genetic models (dominant, recessive, and additive model) were conducted. For each SNP under the additive model, a value of 0 was assigned to wild-type homozygote, 1 to heterozygote, and 2 to variant homozygote. Testing is designed specifically to reveal associations that depend additively on the minor allele. That is, individuals with variant homozygote (as compared with wild-type homozygote) are twice as likely to affect the outcome in a certain direction as individuals with heterozygote (as compared with wild-type homozygote). Similarly, for each SNP under the dominant model, a value of 0 was assigned to wild-type homozygote, 1 to heterozygote, and variant homozygote; while for each SNP under the recessive model, a value of 0 was assigned to wild-type homozygote, and heterozygote, 1 to variant homozygote. Based on results from the logistic regression analyses, SNP allele which increased the risk of GDM was defined as risk allele. All the statistical analyses were performed using Stata Version 11.1 software (Stata, College Station, TX), and *P* < 0.05 in a two-sided test was considered statistically significant. In order to reduce the error caused by multiple comparisons, SNPs with *P* < 0.01 were selected for further detailed analysis. The χ^2^-based *Q*-test was used to evaluate the heterogeneity of associations between subgroups. In addition, statistical power analysis was performed using G^*^Power 3.1.9.2 with an alpha level of 5%.

## Results

### Subject Characteristics

The selected characteristics of the 964 GDM patients and 1,021 controls are shown in Table 1. As expected, there were no significant differences in age and pre-pregnancy BMI between the two groups (*P* = 0.094 and 0.685, respectively). However, there were more multiparaes, women with abnormal pregnancy histories, and women with family histories of diabetes in GDM cases, as compared with the controls (*P* < 0.05 for all comparisons).

**Table 1:** Demographic and selected variables in gestational diabetes cases and controls.

**Variables**	**Gestational** **diabetes cases (*n* = 964) *N* (%)**	**Controls (*n* = 1,021) *N* (%)**	***P***
Age, year (mean ± SD)	30.6 ± 3.7	30.3 ± 3.6	0.094
Pre-pregnancy BMI, kg/m^2^ (mean ± SD)	22.1 ± 2.9	22.0 ± 2.8	0.685
Parity			<0.001
Nulliparae	827 (85.8)	953 (93.3)	
Multiparae	137 (14.2)	68 (6.7)	
Abnormal pregnancy history			<0.001
No	847 (87.9)	981 (96.1)	
Yes	117 (12.1)	40 (3.9)	
Family history of diabetes			0.023
No	791 (82.1)	876 (85.8)	
Yes	173 (17.9)	145 (14.2)	

*SD, standard deviation*.

### The Associations Between Candidate SNPs and GDM Risk

The associations of 33 candidate SNPs with GDM risk were assessed using logistic analyses (Table 2). In the control subjects, the observed frequencies of the 33 SNPs genotype were all at Hardy-Weinberg equilibrium (*P* > 0.05 for all SNPs, Supplementary Table 3). As shown in Table 3, we observed that *FTO* rs1121980 and *KCNQ1* rs163182 were significantly associated with a decreased risk of GDM in the dominant and additive model (dominant model: adjusted OR = 0.78, 95%CI = 0.64–0.94, *P* = 0.011 for rs1121980; adjusted OR = 0.79, 95%CI = 0.65–0.94, *P* = 0.010 for rs163182; additive model: adjusted OR = 0.79, 95%CI = 0.67–0.94, *P* = 0.007 for rs1121980; adjusted OR = 0.84, 95%CI = 0.73–0.96, *P* = 0.009 for rs163182), but not in the recessive model. And *MC4R* rs12970134 and *PROX1* rs340841 were significantly associated with the increased risk of GDM in the three models (dominant model: adjusted OR = 1.22, 95%CI = 1.01–1.47, *P* = 0.040 for rs12970134; adjusted OR =1.33, 95%CI = 1.09–1.63, *P* = 0.005 for rs340841; recessive model: adjusted OR = 1.91, 95%CI = 1.23–2.95, *P* = 0.003 for rs12970134; adjusted OR =1.28, 95%CI = 1.03–1.59, *P* = 0.028 for rs340841; additive model: adjusted OR =1.25, 95%CI = 1.07–1.46, *P* = 0.006 for rs12970134; adjusted OR =1.22, 95%CI = 1.07–1.39, *P* = 0.002 for rs340841).

**Table 2:** Genotyping results in gestational diabetes cases and controls.

**SNP**	**Allele^**a**^**	**Reported gene**	**Chromosome**	**MAF^***b***^**	**Reported MAF^***c***^**	***P*****-value**		
						**Dominant model**	**Recessive model**	**Additive model**
rs11030104	G>A	*BDNF*	11:27662970	0.487	0.223	0.725	0.855	0.745
rs7481311	C>T	*BDNF*	11:27561582	0.265	0.248	0.865	0.867	0.838
rs988712	G>T	*BDNF*	11:27541835	0.142	0.177	0.504	0.297	0.368
rs1121980	G>A	*FTO*	16:53775335	0.188	0.370	0.009	0.075	0.005
rs11642841	C>A	*FTO*	16:53811575	0.039	0.184	0.466	0.501	0.483
rs1421085	T>C	*FTO*	16:53767042	0.122	0.229	0.333	0.573	0.306
rs6499640	G>A	*FTO*	16:53735765	0.158	0.480	0.868	0.472	0.930
rs10278336	A>G	*GCK*	7:44205764	0.365	0.346	0.906	0.510	0.679
rs4607517	G>A	*GCK*	7:44196069	0.233	0.143	0.866	0.811	0.818
rs780093	T>C	*GCKR*	2:27519736	0.486	0.292	0.531	0.337	0.851
rs4846569	C>T	*GCKR*	1:219598379	0.278	0.206	0.915	0.478	0.817
rs163177	T>C	*KCNQ1*	11:2817183	0.473	0.424	0.930	0.681	0.766
rs163182	G>C	*KCNQ1*	11:2822986	0.374	0.398	0.010	0.171	0.011
rs163184	T>G	*KCNQ1*	11:2825839	0.450	0.373	0.520	0.956	0.702
rs2237892	C>T	*KCNQ1*	11:2818521	0.240	0.149	0.114	0.507	0.287
rs2237895	A>C	*KCNQ1*	11:2835964	0.329	0.323	0.730	0.504	0.569
rs2237896	G>A	*KCNQ1*	11:2837210	0.293	0.126	0.142	0.235	0.130
rs2237897	C>T	*KCNQ1*	11:2837316	0.333	0.142	0.746	0.919	0.773
rs231356	A>T	*KCNQ1*	11:2684113	0.191	0.437	0.147	0.011	0.752
rs231362	A>G	*KCNQ1*	11:2670241	0.114	0.271	0.811	0.601	0.941
rs3888647	G>A	*KCNQ1*	11:2932452	0.213	0.306	0.879	0.671	0.783
rs8181588	T>C	*KCNQ1*	11:2810311	0.381	0.193	0.984	0.238	0.531
rs10871777	A>G	*MC4R*	18:60184530	0.201	0.246	0.099	0.583	0.190
rs12970134	G>A	*MC4R*	18:60217517	0.182	0.208	0.036	0.012	0.008
rs476828	T>C	*MC4R*	18:60185354	0.212	0.300	0.133	0.765	0.176
rs538656	G>T	*MC4R*	18:60183189	0.084	0.276	0.102	0.970	0.139
rs1704198	T>G	*PROX1*	1:213737151	0.114	0.114	0.947	0.301	0.845
rs2075423	G>T	*PROX1*	1:213981376	0.186	0.294	0.524	0.264	0.915
rs340841	C>T	*PROX1*	1:213951127	0.444	0.372	0.019	0.031	0.006
rs340874	T>C	*PROX1*	1:213985913	0.380	0.376	0.046	0.803	0.128
rs1496653	A>G	*UBE2E2*	3:23413299	0.242	0.260	0.587	0.669	0.559
rs6780569	G>A	*UBE2E2*	3:23156993	0.203	0.303	0.311	0.998	0.387
rs7612463	C>A	*UBE2E2*	3:23294959	0.208	0.180	0.137	0.409	0.124

a Major > minor allele;

b MAF in 1,021 controls;

c *Reported MAF in Han Chinese from 1,000 genomes. Bold value denotes statistical significance. SNP, single nucleotide polymorphism; MAF, minor allele frequency*.

**Table 3:** Association between four significant SNPs and GDM susceptibility.

**Genotype**	**GDM cases (*n* = 964)** ***N* (%)**	**Controls (*n* = 1,021) *N* (%)**	**OR (95%CI)**	***P***	**OR (95%CI)^**a**^**	***P*^**a**^**
*FTO* rs1121980						
G/G	688 (72.0)	673 (66.5)	1.00		1.00	
A/G	243 (25.4)	298 (29.4)	0.80 (0.65–0.97)	0.027	0.80 (0.65–0.98)	0.028
A/A	25 (2.6)	41 (4.1)	0.60 (0.36–0.99)	0.046	0.62 (0.37–1.04)	0.071
Dominant			0.77 (0.64–0.94)	0.009	0.78 (0.64–0.94)	0.011
Recessive			0.64 (0.38–1.05)	0.075	0.66 (0.40–1.11)	0.112
Additive			0.79 (0.67–0.93)	0.005	0.79 (0.67–0.94)	0.007
*KCNQ1* rs163182						
G/G	427 (45.4)	398 (39.6)	1.00		1.00	
C/G	398 (42.3)	462 (46.0)	0.80 (0.66–0.97)	0.025	0.80 (0.66–0.98)	0.028
C/C	115 (12.2)	144 (14.3)	0.74 (0.56–0.99)	0.039	0.73 (0.55–0.97)	0.030
Dominant			0.79 (0.66–0.94)	0.010	0.79 (0.65–0.94)	0.010
Recessive			0.83 (0.64–1.08)	0.171	0.81 (0.62–1.06)	0.132
Additive			0.85 (0.74–0.96)	0.011	0.84 (0.73–0.96)	0.009
*MC4R* rs12970134						
G/G	590 (62.4)	679 (67.0)	1.00		1.00	
A/G	300 (31.7)	300 (29.6)	1.15 (0.95–1.40)	0.157	1.13 (0.93–1.38)	0.218
A/A	55 (5.8)	35 (3.5)	1.81 (1.17–2.80)	0.008	1.99 (1.28–3.09)	0.002
Dominant			1.22 (1.01–1.47)	0.036	1.22 (1.01–1.47)	0.040
Recessive			1.73 (1.12–2.67)	0.012	1.91 (1.23–2.95)	0.003
Additive			1.23 (1.06–1.44)	0.008	1.25 (1.07–1.46)	0.006
*PROX1* rs340841						
C/C	249 (26.3)	312 (31.1)	1.00		1.00	
C/T	472 (49.8)	492 (49.1)	1.20 (0.98–1.48)	0.084	1.27 (1.02–1.57)	0.030
T/T	226 (23.9)	199 (19.8)	1.42 (1.10–1.83)	0.006	1.49 (1.15–1.92)	0.003
Dominant			1.27 (1.04–1.54)	0.019	1.33 (1.09–1.63)	0.005
Recessive			1.27 (1.02–1.57)	0.031	1.28 (1.03–1.59)	0.028
Additive			1.19 (1.05–1.35)	0.006	1.22 (1.07–1.39)	0.002

a *Logistic regression analyses adjusted for age, pre-pregnancy BMI, parity, abnormal pregnancy history, and family history of diabetes. GDM, gestational diabetes mellitus; SNP, single nucleotide polymorphism*.

Then, we also evaluated the combined effects on GDM by adding the number of risk alleles of the four significant SNPs (rs1121980-G, rs163182-G, rs12970134-A, and rs340841-T). The “0 allele” refers to the subjects carrying rs1121980 AA, rs163182 CC, rs12970134 GG, and rs340841 CC; “1-8 alleles” means 1-8 risk alleles of the four SNPs (rs1121980-G, rs163182-G, rs12970134-A, and rs340841-T). We observed that with the increasing number of the four SNP alleles, GDM risk was significantly increased in a dose-dependent manner (*P*_trend_ < 0.001) (Table 4). When compared with the subjects with“0-3 allele,” subjects carrying “6-8 alleles” had an 84% increase in GDM risk (adjusted OR = 1.84, 95%CI = 1.37–2.47, *P* < 0.001) (Table 4).

**Table 4:** Cumulative effects of the four risk alleles on GDM susceptibility.

**Variables**	**GDM cases** **(*n* = 964) *N* (%)**	**Controls (*n* = 1,021) *N* (%)**	**OR (95%CI)**	***P***	**OR (95%CI)^**a**^**	***P*^**a**^**
Combined effects of *FTO* rs1121980-G, *KCNQ1* rs163182-G, *MC4R* rs12970134-A, and *PROX1* rs340841-T						
0–3	205 (22.5)	293 (29.8)	1.00		1.00	
4–5	540 (59.3)	555 (56.5)	1.39 (1.12–1.72)	0.003	1.43 (1.15–1.78)	0.001
6–8	165 (18.1)	135 (13.7)	1.75 (1.31–2.33)	<0.001	1.84 (1.37–2.47)	<0.001
Trend			*P*^b^ < 0.001		*P*^b^ < 0.001	

a Logistic regression analyses adjusted for age, pre–pregnancy BMI, parity, abnormal pregnancy history, and family history of diabetes.

b *P-value of Cochran-Armitage's trend test. GDM, gestational diabetes mellitus*.

The combined effects of the four SNPs on GDM occurrence were also evaluated by stratifying by age, parity, pre-pregnancy BMI, family history of diabetes, and abnormal pregnancy history. No obvious evidence of heterogeneity associations for the combined effects of the four SNPs on GDM risk was observed (Supplementary Table 4). We also conducted stratified analyses on rs1121980, rs163182, rs12970134, and rs340841 with GDM susceptibility, respectively (Supplementary Table 5). There was also no heterogeneity between the similar association strengths of the subgroups (*P* > 0.05).

### Genetic Risk Score Calculation

Then, a genetic risk score (GRS) was calculated based on the number of risk alleles (*FTO* rs1121980-G, *KCNQ1* rs163182-G, *MC4R* rs12970134-A, and *PROX1* rs340841-T) an individual inherits. The natural ln of each OR for each SNP was multiplied by the number of risk alleles (2, 1, or 0) to generate a genotypic OR; said values for each locus were then summed. GDM risk as a function of GRS in our population was examined in Table 5. The mean GRS for cases was significantly higher than that calculated for the controls (*P* < 0.001). An analysis of risk by quartile of GRS indicated that individuals with the highest quartile of GRS having a 1.94 fold risk of GDM, when compared to the individuals with the lowest quartile of GRS.

**Table 5:** Risk stratification for GDM using genetic risk score.

**Quartile**	**Risk score**	**GDM cases** **(*n* = 964) *N* (%)**	**Controls (*n* = 1,021) *N* (%)**	**OR (95%CI)**	***P***	**OR (95%CI)^**a**^**	***P*^**a**^**
0	0~	194 (21.3)	283 (28.8)	1.00		1.00	
1	0.6555~	239 (26.3)	259 (26.3)	1.35 (1.05–1.74)	0.022	1.39 (1.07–1.80)	0.013
2	0.8227~	230 (25.3)	247 (25.1)	1.36 (1.05–1.76)	0.019	1.41 (1.08–1.83)	0.011
3	1.0101~	247 (27.1)	194 (19.7)	1.86 (1.43–2.41)	<0.001	1.94 (1.48–2.53)	<0.001
	Trend			*P*^b^ < 0.001		*P*^b^ < 0.001	

a Logistic regression analyses adjusted for age, pre-pregnancy BMI, parity, abnormal pregnancy history, and family history of diabetes.

b *P-value of Cochran-Armitage's trend test. GDM, gestational diabetes mellitus*.

### Functional Annotation of the Significant SNPs

In the databases of ENCODE and Roadmap, the potential functions of the four significant SNPs (rs1121980, rs163182, rs12970134, and rs340841) were explored. These public databases showed that the four significant SNPs were all fell in the functional elements of the related genes in the human pancreatic islets, including Transcription Factor ChIP-seq Clusters, DNaseI hypersensitivity (DNaseI HS) density signal, Formaldehyde-Assisted Isolation of Regulatory Elements (FAIRE) density signal, or histone modification markers, such as H3K27ac, H3K27me3, H3K36me3, H3K4me1, H3K4me3, and H3K9me3 (Supplementary Figures 2–5). We further analyzed the genotype-phenotype correlations by exploiting the PhenoScanner database. We found *FTO* rs1121980 and its correlated variants within an LD block were significantly associated with FTO expression in muscle skeletal and whole blood (Supplementary Table 6). For rs163182 and rs12970134, although their relationship with KCNQ1 and MC4R are not present, the two SNPs and their correlated variants were significantly associated with the expression of multiple genes in several tissues (Supplementary Tables 7,8). In addition, *PROX1* rs340841 and its correlated variants were significantly associated with PROX1-AS1 expression in pancreas and multiple other tissues (Supplementary Table 9).

### Power Analysis

We calculated the statistical power analysis to reassess the available data when an alpha of 0.05 was assigned. For *FTO* rs1121980 analysis, the statistical power ranged from 14.4 to 99.9%; for *KCNQ1* rs163182, the statistical power ranged from 11.5 to 89.1%; for *MC4R* rs12970134, the statistical power ranged from 12.2 to 41.6%; for *PROX1* rs340841, the statistical power ranged from 6.9 to 45.0%, and for the combined effects of the four risk alleles and stratified analyses, the statistical power ranged from 7.3 to 99.9%.

## Discussion

Limited but increasing evidences suggested that similar genetic risk variants which predispose to T2D and obesity also contribute to the risk of GDM (9). In this case-control study, we investigated the association of 33 thus far published confirmed genetic variants for obesity or metabolic diseases with GDM in a Chinese population. Our study showed that several risk variants for obesity or metabolic diseases (*FTO* rs1121980, *KCNQ1* rs163182, *MC4R* rs12970134, and *PROX1* rs340841) were also associated with GDM, giving further evidence that GDM and obesity/metabolic diseases may share a similar genetic background. We observed that *FTO* rs1121980 and *KCNQ1* rs163182 conferred a decreased GDM risk, whereas *MC4R* rs12970134 and *PROX1* rs340841 conferred an increased GDM risk. With the increasing number of risk alleles of the four significant SNPs, GDM risk was significantly increased in a dose-dependent manner. And the significant positive associations between the weighted genetic risk score and risk of GDM persisted, suggesting that *FTO* rs1121980, *KCNQ1* rs163182, *MC4R* rs12970134, and *PROX1* rs340841 may be markers for susceptibility to GDM in a Chinese population.

*FTO* gene is located in chromosome 16q12.2, reported to be consistently related to obesity and BMI (10). It is highly expressed in hypothalamic nuclei and extensively expressed in brain, playing an important role in the energy balance (19). Given the connection with obesity, many studies have been conducted to identify the association between genetic variants in *FTO* and risk of T2D/GDM (11). In the early GWA study and subsequent validated studies, the authors suggested that *FTO* rs1121980 may represent a susceptibility locus for obesity risk (20, 21). Its association with T2D was also reported (22). The effects of *FTO* polymorphisms on T2D susceptibility may be mediated through their effect on increasing the lifetime maximum BMI before or at the time of diagnosis (23). However, in two meta-analysis on GDM, no associations between *FTO* polymorphisms (rs9939609, rs8050136, rs1421085, rs9939609, and rs8050136) and GDM risk were found (24, 25). Our results also indicated a lack of association between *FTO* rs1421085 and GDM risk, but we found that *FTO* rs1121980 was significantly associated with decreased GDM risk (OR = 0.79, *P* = 0.007), which added new evidence for the association of *FTO* polymorphisms with GDM.

The association of *KCNQ1* rs163182 with GDM was also firstly reported in this study. *KCNQ1* is a gene that wildly expressed in cardiac muscle, inner ear, kidney, lung, stomach, and intestine, providing instructions for making potassium channels. It is also expressed in pancreas, and could affect the insulin secretion and insulin sensitivity (26). The gene of *KCNQ1* not only plays an important role in blood glucose metabolism but also regulates other metabolic substances (27, 28). It has been confirmed that the KCNQ1 gene was associated with diabetes, metabolic syndrome, and lipid parameters (29, 30). In a GWA study for T2D conducted in southern China (31), the authors confirmed the association between *KCNQ1* rs163182 and T2D. So, our result that *KCNQ1* rs163182 conferred a decreased GDM risk was understood. Previous association study and meta-analysis also reported that *KCNQ1* rs2074196, rs2237892, and rs2237895 were associated with the risk of GDM (32–34). Based on the causal inference (35), GDM was a risk factor for T2D and the KCNQ1 gene was associated with T2D and GDM. Thus, the KCNQ1 gene may influence GDM occurrence.

MC4R is a G protein-coupled receptor which is expressed in the hypothalamus and implicated in the energy balance regulation (36). MC4R has been associated with key components of appetite, food intake, nutrient absorption, thermogenesis, energy expenditure, insulin secretion, obesity, and lipid metabolism (37). It is indicated that *MC4R* rs6567160 is associated with postpartum weight reduction and glycemic changes among women with prior GDM (38). Moreover, a significant correlation was observed between lipid parameters and *MC4R* rs17782313 (39). In the previous GWA study, the authors identified rs12970134 near *MC4R* associated with waist circumference and insulin resistance (40). And this variant was also associated with obesity and T2D (41, 42). In this study, we put forward *MC4R* rs12970134 conferred an increased GDM risk, further confirming the genetic similarity between GDM and obesity or metabolic diseases.

PROX1 encodes the prospero homeobox 1 protein, which is a homeobox transcription factor involved in developmental processes of multiple organs, such as progenitor cell regulation, gene transcription regulation, and cell fate determination. PROX1 gene also plays an important role in the embryo development (43). And PROX1 has been shown to be associated with diabetes and its complications in a number of studies (43, 44). Recently, a GWA study revealed that *PROX1* rs340841 is a strong susceptibility locus of early onset of diabetes with variations depending on ethnicity (45). Here, we present evidence that *PROX1* rs340841 conferred an increased GDM risk in a Chinese population. It is of interest that several polymorphisms at this locus are associated with insulin levels.

In this study, GDM cases and controls were all selected from a population-based, large study for systematic screening of pregnancy complications, and the two groups were matched well-according to age and pre-pregnancy BMI, which may help reduce potential selection bias. However, several limitations ought to be acknowledged. Firstly, restricted by the conditions, the number of related patients and controls in this study is relatively small. Especially in subgroups, statistical power may be limited to finding differences between the groups. Secondly, some GDM related phenotypes, such as fasting plasma gluocose, HbA1c, 2 h Plasma glucose, insulin levels, were not obtained. So the associations of the significant SNPs with these phenotypes were not analyzed. Further quantitative studies for the GDM related phenotypes are needed. Thirdly, some reported GDM risk factors, such as weight gain during pregnancy, exercise and diet were not adjusted in the statistical analyses for the lack of related data. Fourthly, most associations reported were not reach significance after multiple comparison correction, which was another drawback of this study. Therefore, confirmation from additional patients in the further studies is warranted.

## Conclusions

Taken together, our study demonstrated for the first time that *FTO* rs1121980, *KCNQ1* rs163182, *MC4R* rs12970134, and *PROX1* rs340841 were associated with risk of GDM in Chinese population. Further studies conducted in different populations with functional assays are needed to validate our findings, and to investigative the predictability of these genetic variants for the development of metabolic related diseases later in life in those with GDM.

## Data Availability Statement

All datasets generated for this study are included in the article/Supplementary Files.

## Ethics Statement

All subjects gave written informed consent. This study was approved by the ethics committee of Women's Hospital of Nanjing Medical University (NFY201608), and all methods were carried out from August 2016 until December 2018, in accordance with the 1964 Principles of the Helsinki Declaration and its later amendments.

## Author Contributions

MC conceived and designed the idea, did data collection, wrote and drafted the manuscript. LZ, TC, and AS did data collection. KX did literature review. ZL and JX reviewed the manuscript. ZC performed the data analysis. JW and CJ designed and contributed to the reviewing of the final manuscript. All authors approved the final format of the submitted manuscript.

## Conflict of Interest

The authors declare that the research was conducted in the absence of any commercial or financial relationships that could be construed as a potential conflict of interest.

## Supplementary Material

Table S1 Initial list of variants from GWA study selected for genotyping.

## References

[1] Lavery JA Friedman AM Keyes KM Wright JD Ananth CV. Gestational diabetes in the United States: temporal changes in prevalence rates between 1979 and 2010. BJOG. (2017) 124:804–13. 10.1111/1471-0528.14236 27510598 PMC5303559

[2] Zhu Y Zhang C. Prevalence of gestational diabetes and risk of progression to type 2 diabetes: a global perspective. Curr Diab Rep. (2016) 16:7. 10.1007/s11892-015-0699-x 26742932 PMC6675405

[3] Billionnet C Mitanchez D Weill A Nizard J Alla F Hartemann A. Gestational diabetes and adverse perinatal outcomes from 716,152 births in France in 2012. Diabetologia. (2017) 60:636–44. 10.1007/s00125-017-4206-6 28197657 PMC6518373

[4] McIntyre HD Catalano P Zhang C Desoye G Mathiesen ER Damm P. Gestational diabetes mellitus. Nat Rev Dis Primers. (2019) 5:47. 10.1038/s41572-019-0098-8 31296866

[5] Johns EC Denison FC Norman JE Reynolds RM. Gestational diabetes mellitus: mechanisms, treatment, and complications. Trends Endocrinol Metab. (2018) 29:743–54. 10.1016/j.tem.2018.09.004 30297319

[6] Kwak SH Kim SH Cho YM Go MJ Cho YS Choi SH. A genome-wide association study of gestational diabetes mellitus in Korean women. Diabetes. (2012) 61:531–41. 10.2337/db11-1034 22233651 PMC3266417

[7] Wu L Cui L Tam WH Ma RC Wang CC. Genetic variants associated with gestational diabetes mellitus: a meta-analysis and subgroup analysis. Sci Rep. (2016) 6:30539. 10.1038/srep30539 27468700 PMC4965817

[8] Ding M Chavarro J Olsen S Lin Y Ley SH Bao W. Genetic variants of gestational diabetes mellitus: a study of 112 SNPs among 8722 women in two independent populations. Diabetologia. (2018) 61:1758–68. 10.1007/s00125-018-4637-8 29947923 PMC6701842

[9] Konig M Shuldiner AR. The genetic interface between gestational diabetes and type 2 diabetes. J Matern Fetal Neonatal Med. (2012) 25:36–40. 10.3109/14767058.2012.626926 22145702

[10] Tung YCL Yeo GSH O'Rahilly S Coll AP Obesity and CTO: changing focus at a complex locus. Cell Metab. (2014) 20:710–8. 10.1016/j.cmet.2014.09.010 25448700

[11] Lin Z Wang Y Zhang B Jin Z. Association of type 2 diabetes susceptible genes GCKR, SLC30A8, and FTO polymorphisms with gestational diabetes mellitus risk: a meta-analysis. Endocrine. (2018) 62:34–45. 10.1007/s12020-018-1651-z 30091126

[12] Angueira AR Ludvik AE Reddy TE Wicksteed B Lowe WL Jr. Layden BT. New insights into gestational glucose metabolism: lessons learned from 21st century approaches. Diabetes. (2015) 64:327–34. 10.2337/db14-0877 25614666 PMC4876793

[13] Palmer ND Goodarzi MO Langefeld CD Wang N Guo X Taylor KD. Genetic variants associated with quantitative glucose homeostasis traits translate to type 2 diabetes in Mexican Americans: the GUARDIAN (Genetics Underlying Diabetes In Hispanics) consortium. Diabetes. (2015) 64:1853–66. 10.2337/db14-0732 25524916 PMC4407862

[14] Imamura M Takahashi A Yamauchi T Hara K Yasuda K Grarup N. Genome-wide association studies in the Japanese population identify seven novel loci for type 2 diabetes. Nat Commun. (2016) 7:10531. 10.1038/ncomms10531 26818947 PMC4738362

[15] Berndt SI Gustafsson S Magi R Ganna A Wheeler E Feitosa MF. Genome-wide meta-analysis identifies 11 new loci for anthropometric traits and provides insights into genetic architecture. Nat Genet. (2013) 45:501–12. 10.1038/ng.2606 23563607 PMC3973018

[16] Tsai FJ Yang CF Chen CC Chuang LM Lu CH Chang CT. A genome-wide association study identifies susceptibility variants for type 2 diabetes in Han Chinese. PLoS Genet. (2010) 6:e1000847. 10.1371/journal.pgen.1000847 20174558 PMC2824763

[17] Su T Ren Q Lu Y Tai W Zhu Y Li Z. A genetic variant in LINGO2 contributes to the risk of gestational diabetes mellitus in a Chinese population. J Cell Physiol. (2019) 234:7012–8. 10.1002/jcp.27454 30426492

[18] Hoffman L Nolan C Wilson JD Oats JJ Simmons D. Gestational diabetes mellitus–management guidelines. The Australasian diabetes in pregnancy society. Med J Aust. (1998) 169:93–7. 9700346 10.5694/j.1326-5377.1998.tb140192.x

[19] Merkestein M Laber S McMurray F Andrew D Sachse G Sanderson J. FTO influences adipogenesis by regulating mitotic clonal expansion. Nat Commun. (2015) 6:6792. 10.1038/ncomms7792 25881961 PMC4410642

[20] Peng S Zhu Y Xu F Ren X Li X Lai M. FTO gene polymorphisms and obesity risk: a meta-analysis. BMC Med. (2011) 9:71. 10.1186/1741-7015-9-71 21651756 PMC3118373

[21] Hinney A Nguyen TT Scherag A Friedel S Bronner G Muller TD. Genome Wide Association (GWA) study for early onset extreme obesity supports the role of fat mass and obesity associated gene (FTO) variants. PLoS ONE. (2007) 2:e1361. 10.1371/journal.pone.0001361 18159244 PMC2137937

[22] Zhu Z Tong X Zhu Z Liang M Cui W Su K. Development of GMDR-GPU for gene-gene interaction analysis and its application to WTCCC GWAS data for type 2 diabetes. PLoS ONE. (2013) 8:e61943. 10.1371/journal.pone.0061943 23626757 PMC3633958

[23] Kamura Y Iwata M Maeda S Shinmura S Koshimizu Y Honoki H. FTO gene polymorphism is associated with type 2 diabetes through its effect on increasing the maximum BMI in Japanese men. PLoS ONE. (2016) 11:e0165523. 10.1371/journal.pone.0165523 27820839 PMC5098825

[24] He H Cao WT Zeng YH Huang ZQ Du WR Guan ND. Lack of associations between the FTO polymorphisms and gestational diabetes: a meta-analysis and trial sequential analysis. Gene. (2018) 677:169–75. 10.1016/j.gene.2018.07.064 30055308

[25] Guo F Long W Zhou W Zhang B Liu J Yu B. FTO, GCKR, CDKAL1 AND CDKN2A/B gene polymorphisms and the risk of gestational diabetes mellitus: a meta-analysis. Arch Gynecol Obstet. (2018) 298:705–15. 10.1007/s00404-018-4857-7 30074065

[26] Yamagata K Senokuchi T Lu M Takemoto M Fazlul Karim M Go C. Voltage-gated K+ channel KCNQ1 regulates insulin secretion in MIN6 beta-cell line. Biochem Biophys Res Commun. (2011) 407:620–5. 10.1016/j.bbrc.2011.03.083 21426901

[27] Boini KM Graf D Hennige AM Koka S Kempe DS Wang K. Enhanced insulin sensitivity of gene-targeted mice lacking functional KCNQ1. Am J Physiol Regul Integr Comp Physiol. (2009) 296:R1695–701. 10.1152/ajpregu.90839.2008 19369585 PMC2692790

[28] Mussig K Staiger H Machicao F Kirchhoff K Guthoff M Schafer SA. Association of type 2 diabetes candidate polymorphisms in KCNQ1 with incretin and insulin secretion. Diabetes. (2009) 58:1715–20. 10.2337/db08-1589 19366866 PMC2699873

[29] Gao K Wang J Li L Zhai Y Ren Y You H. Polymorphisms in four genes (KCNQ1 RS151290, KLF14 RS972283, GCKR RS780094 AND MTNR1B RS10830963) and their correlation with type 2 diabetes mellitus in Han Chinese in Henan Province, China. Int J Environ Res Public Health. (2016) 13:260. 10.3390/ijerph13030260 26927145 PMC4808923

[30] Liu Y Wang C Chen Y Yuan Z Yu T Zhang W. A variant in KCNQ1 gene predicts metabolic syndrome among northern urban Han Chinese women. BMC Med Genet. (2018) 19:153. 10.1186/s12881-018-0652-3 30157802 PMC6114251

[31] Cui B Zhu X Xu M Guo T Zhu D Chen G. A genome-wide association study confirms previously reported loci for type 2 diabetes in Han Chinese. PLoS ONE. (2011) 6:e22353. 10.1371/journal.pone.0022353 21799836 PMC3142153

[32] Ao D Wang HJ Wang LF Song JY Yang HX Wang Y. The RS2237892 polymorphism in KCNQ1 influences gestational diabetes mellitus and glucose levels: a case-control study and meta-analysis. PLoS ONE. (2015) 10:e0128901. 10.1371/journal.pone.0128901 26039078 PMC4454508

[33] Mao H Li Q Gao S. Meta-analysis of the relationship between common type 2 diabetes risk gene variants with gestational diabetes mellitus. PLoS ONE. (2012) 7:e45882. 10.1371/journal.pone.0045882 23029294 PMC3454322

[34] Kwak SH Kim TH Cho YM Choi SH Jang HC Park KS. Polymorphisms in kCNQ1 are associated with gestational diabetes in a Korean population. Horm Res Paediatr. (2010) 74:333–8. 10.1159/000313918 20606385

[35] Pearl J Causal inference in statistics: an overview. Stat Surv. (2009) 3:96–46. 10.1214/09-ss057

[36] Rossi J Balthasar N Olson D Scott M Berglund E Lee CE. Melanocortin-4 receptors expressed by cholinergic neurons regulate energy balance and glucose homeostasis. Cell Metab. (2011) 13:195–204. 10.1016/j.cmet.2011.01.010 21284986 PMC3033043

[37] Adan RA Tiesjema B Hillebrand JJ la Fleur SE Kas MJ de Krom M. The MC4 receptor and control of appetite. Br J Pharmacol. (2006) 149:815–27. 10.1038/sj.bjp.0706929 17043670 PMC2014686

[38] de Carvalho AM Shao P Liu H Cheng HL Zheng Y Leng J. The MC4R genotype is associated with postpartum weight reduction and glycemic changes among women with prior gestational diabetes: longitudinal analysis. Sci Rep. (2017) 7:9654. 10.1038/s41598-017-10101-x 28852042 PMC5575005

[39] Franzago M Fraticelli F Nicolucci A Celentano C Liberati M Stuppia L. Molecular analysis of a genetic variants panel related to nutrients and metabolism: association with susceptibility to gestational diabetes and cardiometabolic risk in affected women. J Diabetes Res. (2017) 2017:4612623. 10.1155/2017/4612623 28133617 PMC5241477

[40] Chambers JC Elliott P Zabaneh D Zhang W Li Y Froguel P. Common genetic variation near MC4R is associated with waist circumference and insulin resistance. Nat Genet. (2008) 40:716–8. 10.1038/ng.156 18454146

[41] Kong X Zhang X Zhao Q He J Chen L Zhao Z. Obesity-related genomic loci are associated with type 2 diabetes in a Han Chinese population. PLoS ONE. (2014) 9:e104486. 10.1371/journal.pone.0104486 25093408 PMC4122466

[42] Yang Y Gao X Tao X Gao Q Zhang Y Yang J. Combined effect of FTO and MC4R gene polymorphisms on obesity in children and adolescents in Northwest China: a case-control study. Asia Pac J Clin Nutr. (2019) 28:177–82. 10.6133/apjcn.201903_28(1).0023 30896429

[43] Lecompte S Pasquetti G Hermant X Grenier-Boley B Gonzalez-Gross M de Henauw S. Genetic and molecular insights into the role of PROX1 in glucose metabolism. Diabetes. (2013) 62:1738–45. 10.2337/db12-0864 23274905 PMC3636631

[44] Dupuis J Langenberg C Prokopenko I Saxena R Soranzo N Jackson AU. New genetic loci implicated in fasting glucose homeostasis and their impact on type 2 diabetes risk. Nat Genet. (2010) 42:105–16. 10.1038/ng.520 20081858 PMC3018764

[45] Hamet P Haloui M Harvey F Marois-Blanchet FC Sylvestre MP Tahir MR. PROX1 gene CC genotype as a major determinant of early onset of type 2 diabetes in slavic study participants from action in diabetes and vascular disease: preterax and diamicron MR controlled evaluation study. J Hypertens. (2017) 35(Suppl. 1):S24–32. 10.1097/HJH.0000000000001241 28060188 PMC5377997

