# Supplementary material for: Genetic Susceptibility to Gestational Diabetes Mellitus in a Chinese Population
Source: Front Endocrinol (Lausanne). 2020 Apr 22;11:247. doi: 10.3389/fendo.2020.00247 (PMC7188786; doi:10.3389/fendo.2020.00247)
Supplement: Supplementary file 2 [file Data_Sheet_2.docx]

**Supplementary Tables 2-9**

| **Supplementary Table 2. Information of primers for Sequenom MassARRAY iPLEX assays** | | | |
| --- | --- | --- | --- |
| SNP_ID | 2nd-PCRP | 1st-PCRP | UEP_SEQ |
| rs11030104 | ACGTTGGATGCACAGGAAATTGTAGGACAG | ACGTTGGATGTGACCAACTTCTTGAGTTCC | GGAAATTGTAGGACAGTTAGTA |
| rs7481311 | ACGTTGGATGAACTATCTCAGCCTTTGAGC | ACGTTGGATGCTCCTGAACCCCTTACTATG | cGCCTTTGAGCCCAACA |
| rs988712 | ACGTTGGATGTCTTCTTAGCAGATTGCAAG | ACGTTGGATGGGTATATAAAGCTGATTCTGG | tcTTAACTATTGTCCCTATGCTATA |
| rs1121980 | ACGTTGGATGCTTATATGGCCCCACCTTTC | ACGTTGGATGCAAGAGTTACAGGTAGGCAG | ctccgAGTCACGTGTCTTGGTACTAT |
| rs11642841 | ACGTTGGATGAGTCCTCTTATAGGTCTGTC | ACGTTGGATGGTTGCCATGCTCAGATTTAC | GTTTTCCTCTTTGCCCC |
| rs1421085 | ACGTTGGATGGTAGCAGTTCAGGTCCTAAG | ACGTTGGATGGGAGACTACCCTACAAATTC | ccatcTCAGGTCCTAAGGCATGA |
| rs6499640 | ACGTTGGATGAGTTGGATAAGCTTTCTGCC | ACGTTGGATGGAACTGATGGTAGAGTATTTC | tAACAGGGTTTCTCTGAA |
| rs10278336 | ACGTTGGATGAGCACTGTGCTAAAATCCTC | ACGTTGGATGGAACAATCCTGTGGATTGTG | cctccAATCCTCATATGATTGAAAGG |
| rs4607517 | ACGTTGGATGACCAAAAAGCAAGACCTGCC | ACGTTGGATGTTGTGATGTGTCAGTGCTGG | CGCCCCAGCTGTCAC |
| rs780093 | ACGTTGGATGATGCATGTGTCCCAAGCAAG | ACGTTGGATGTACGTAGCAACACTAGTGGG | gCCCAAGCAAGAGCCCCC |
| rs4846569 | ACGTTGGATGGTCTCTAAATGGTGTTTGTG | ACGTTGGATGGGGACCAAAAATGACTGTTC | gATGGTGTTTGTGTTGAAAAGAA |
| rs163177 | ACGTTGGATGTCTGCGCACGCTCCTTTCAA | ACGTTGGATGTTTCATGTCAAAGGCAAGCG | CTTTCAAGGGTTAACGG |
| rs163182 | ACGTTGGATGATCTCCCTGAAAGTGGAATG | ACGTTGGATGGGATGTCCTTGGATTGAGTC | gccaTGAAAGTGGAATGAAGACT |
| rs163184 | ACGTTGGATGTTGCCCTGCCTCTCTCTTTA | ACGTTGGATGGGTACTTTGCTCAGTAACGG | GCCTCTCTCTTTACTCCAA |
| rs2237892 | ACGTTGGATGTGTAAGGCATCTGGTGGAGA | ACGTTGGATGCAGATGATGGGAGCTGTCAC | aagatGGGTTTCTAGGCCCCTCACCCC |
| rs2237895 | ACGTTGGATGAGATGACAGGGCAGTGACAG | ACGTTGGATGCTCCTATGACTTCGATTCCC | cccGCCCGGTCAGTGGTCCC |
| rs2237896 | ACGTTGGATGTGCTGTGTTCTGTCTCCACC | ACGTTGGATGAGAGAGGACACCGTCTTGAG | agCCACCCAGGCCTCTCCC |
| rs2237897 | ACGTTGGATGTGGCCAATGTTGAGGGACG | ACGTTGGATGGCTCAGGGGAAGGATGAGG | tgggGCCCAGGGAGCTGGGGA |
| rs231356 | ACGTTGGATGGTACAGCAATTTCAGAACCC | ACGTTGGATGCCCATTCTTCAAGGGAAACG | ttatgTCAGAACCCTTTCACATAG |
| rs231362 | ACGTTGGATGATGGGTTGCCTAGAGACAAG | ACGTTGGATGGTAGCTCACCTGCCTTTGAC | TTCCCTCGCCCGTCA |
| rs3888647 | ACGTTGGATGTACCACCACCACTAACTCAC | ACGTTGGATGTCAGGCCTCAGGCAAGGGA | ACCACTAACTCACACTGCC |
| rs8181588 | ACGTTGGATGTACAGAGACCTTTCCAGAGC | ACGTTGGATGGTTCATTGACTGTTCTGGGC | CCAGAGCTCAAGGACT |
| rs10871777 | ACGTTGGATGTGAAAACAGCACAGTCACAC | ACGTTGGATGACGCGAACACACCTATATTG | CAGTCACACACATGACA |
| rs12970134 | ACGTTGGATGCGGTTCTAAGCAACAGATAC | ACGTTGGATGACAGGTACTAACAAGCACCC | cCTTACCAAACAAAGCATGA |
| rs476828 | ACGTTGGATGAGCAGCACCTGAGTAAAGAG | ACGTTGGATGGATTCTGAGAGATAAGATTG | AAACTGTGGGGTGTC |
| rs538656 | ACGTTGGATGATGTAGAGCTTTAACTCACC | ACGTTGGATGTTTAGCTATTATAGATATATG | AGCTTTAACTCACCATAATTTTG |
| rs1704198 | ACGTTGGATGGCTGAATTCTGCCCCTAAGT | ACGTTGGATGCCATGGCCACAGGTTGATAC | ggcgGCCCCTAAGTTTTTTACAGA |
| rs2075423 | ACGTTGGATGCTCTCCTTCTTGTTGTTCCC | ACGTTGGATGGCATCTCCTAAAGAAACCGC | ccTGTCCTGTGGTTTCATT |
| rs340841 | ACGTTGGATGGAAAGCATGGCTACATGTTC | ACGTTGGATGTGCTGTAAAGGTGGCTAGTC | catggTCCCTTGAACATCAAGTGAA |
| rs340874 | ACGTTGGATGGAGCAGATGGTTTTAAGGTG | ACGTTGGATGCATATAAGTTAGCGCCAGCC | cccggTTTAAGGTGTGGAAAGGTATA |
| rs1496653 | ACGTTGGATGGCTCTACTGCTGCAGAAAAC | ACGTTGGATGGCACATCGTATGGTTAACAC | ACAAATGTGGTATGTATTTCA |
| rs6780569 | ACGTTGGATGTCTTTTTTCTCCCTCCTTGC | ACGTTGGATGTTGGCAGGGTGATAAAAGGG | CTTGCATTTATTTTCTTCCCTC |
| rs7612463 | ACGTTGGATGGAGGAAGTAGAGAGAAGTGG | ACGTTGGATGCTGCCTAATACAGGGTCTTC | GTTTTAGACTCAGTTTCAGGTA |
| Note: SNP, single-nucleotide polymorphism; PCRP, primer for polymerase chain reaction; UEP_SEQ, primer for single nucleotide extension. | | | |

| **Supplementary Table 3. *P* values for Hardy-Weinberg equilibrium** | | | |
| --- | --- | --- | --- |
| SNP_ID | *P* | SNP_ID | *P* |
| rs11030104 | 0.083 | rs2237897 | 0.836 |
| rs7481311 | 0.122 | rs231356 | 0.311 |
| rs988712 | 0.392 | rs231362 | 0.209 |
| rs1121980 | 0.822 | rs3888647 | 0.774 |
| rs11642841 | 0.679 | rs8181588 | 0.551 |
| rs1421085 | 0.373 | rs10871777 | 0.065 |
| rs6499640 | 0.085 | rs12970134 | 0.919 |
| rs10278336 | 0.089 | rs476828 | 0.712 |
| rs4607517 | 0.239 | rs538656 | 0.623 |
| rs780093 | 0.608 | rs1704198 | 0.272 |
| rs4846569 | 0.061 | rs2075423 | 0.091 |
| rs163177 | 0.750 | rs340841 | 0.750 |
| rs163182 | 0.890 | rs340874 | 0.501 |
| rs163184 | 0.702 | rs1496653 | 0.104 |
| rs2237892 | 0.678 | rs6780569 | 0.508 |
| rs2237895 | 0.269 | rs7612463 | 0.131 |
| rs2237896 | 0.274 |  |  |

| **Supplementary Table 4. Stratified analyses on the combined effects of the four risk alleles with GDM susceptibility** | | | | | | |
| --- | --- | --- | --- | --- | --- | --- |
| Variables | 0-3 / 4-5 / 6-8 allele (s) | | OR (95%CI) | *P* ^b^ | OR (95%CI) ^a^ | *P* ^b^ |
|  | GDM cases (n = 964) N (%) | Controls (n = 1021) N (%) |  |  |  |  |
| Age, year |  |  |  |  |  |  |
| <= 30 | 96 (20.9) / 276 (60.1) / 87 (19.0) | 182 (30.1) / 338 (55.9) / 85 (14.0) | 1.41 (1.16-1.71) | 0.447 | 1.46 (1.20-1.79) | 0.382 |
| > 30 | 109 (24.2) / 264 (58.5) / 78 (17.3) | 110 (29.3) / 217 (57.7) / 49 (13.0) | 1.26 (1.02-1.57) |  | 1.28 (1.03-1.59) |  |
| Pre-pregnancy BMI, kg/m^2^ | |  |  |  |  |  |
| <= 22 | 109 (23.0) / 285 (60.1) / 80 (16.9) | 158 (31.2) / 285 (56.3) / 63 (12.5) | 1.37 (1.12-1.68) | 0.678 | 1.43 (1.16-1.75) | 0.590 |
| > 22 | 96 (22.0) / 255 (58.5) / 85 (19.5) | 135 (28.3) / 270 (56.6) / 72 (15.1) | 1.29 (1.06-1.58) |  | 1.32 (1.08-1.63) |  |
| Parity |  |  |  |  |  |  |
| Nulliparae | 175 (22.5) / 461 (59.3) / 141 (18.1) | 272 (29.7) / 520 (56.8) / 124 (13.5) | 1.34 (1.15-1.55) | 0.852 | 1.38 (1.18-1.61) | 0.546 |
| Multiparae | 30 (22.6) / 79 (59.4) / 24 (18.0) | 21 (31.3) / 35 (52.2) / 11 (16.4) | 1.28 (0.81-2.02) |  | 1.17 (0.70-1.95) |  |
| Abnormal pregnancy history | |  |  |  |  |  |
| No | 173 (21.7) / 475 (59.7) / 148 (18.6) | 279 (29.5) / 533 (56.4) / 133 (14.1) | 1.35 (1.17-1.57) | 0.579 | 1.36 (1.17-1.58) | 0.538 |
| Yes | 32 (28.1) / 65 (57.0) / 17 (14.9) | 14 (36.8) / 22 (57.9) / 2 (5.3) | 1.61 (0.88-2.95) |  | 1.69 (0.86-3.32) |  |
| Family history of diabetes | |  |  |  |  |  |
| No | 162 (21.8) / 447 (60.1) / 135 (18.1) | 253 (30.0) / 476 (56.5) / 114 (13.5) | 1.37 (1.18-1.61) | 0.343 | 1.41 (1.20-1.66) | 0.300 |
| Yes | 43 (25.9) / 93 (56.0) / 30 (18.1) | 40 (28.6) / 79 (56.4) / 21 (15.0) | 1.14 (0.81-1.62) |  | 1.15 (0.81-1.63) |  |
| Note: ^a^ Logistic regression analyses adjusted for age, pre-pregnancy BMI, parity, abnormal pregnancy history and family history of diabetes (excluded the stratified factor in each stratum). ^b^ *P* -value for the heterogeneity test. Abbreviation: GDM, gestational diabetes mellitus. | | | | | | |

| **Supplementary Table 5. Stratified analyses on the association between the four significant SNPs and GDM risk, respectively** | | | | | | | | |
| --- | --- | --- | --- | --- | --- | --- | --- | --- |
| Variables | *FTO* rs1121980 GG/AG/AA | | *KCNQ1* rs163182 GG/CG/CC | | *MC4R* rs12970134 GG/AG/AA | | *PROX1* rs340841 CC/CT/TT | |
|  | OR (95%CI) | *P* ^a^ | OR (95%CI) | *P* ^a^ | OR (95%CI) | *P* ^a^ | OR (95%CI) | *P* ^a^ |
| Age, year |  |  |  |  |  |  |  |  |
| <= 30 | 0.72 (0.58-0.91) | 0.298 | 0.81 (0.68-0.96) | 0.596 | 1.28 (1.04-1.58) | 0.608 | 1.20 (1.01-1.42) | 0.846 |
| > 30 | 0.86 (0.67-1.10) |  | 0.87 (0.71-1.06) |  | 1.18 (0.94-1.49) |  | 1.17 (0.97-1.42) |  |
| Pre-pregnancy BMI, kg/m^2^ | |  |  |  |  |  |  |  |
| <= 22 | 0.79 (0.63-0.99) | 1.000 | 0.82 (0.69-0.99) | 0.653 | 1.16 (0.93-1.44) | 0.437 | 1.24 (1.04-1.48) | 0.560 |
| > 22 | 0.79 (0.62-1.00) |  | 0.87 (0.72-1.04) |  | 1.31 (1.06-1.63) |  | 1.15 (0.96-1.38) |  |
| Parity |  |  |  |  |  |  |  |  |
| Nulliparae | 0.81 (0.68-0.97) | 0.448 | 0.85 (0.74-0.98) | 0.652 | 1.22 (1.03-1.43) | 0.746 | 1.24 (1.09-1.42) | 0.090 |
| Multiparae | 0.65 (0.38-1.12) |  | 0.77 (0.51-1.15) |  | 1.34 (0.78-2.31) |  | 0.87 (0.59-1.28) |  |
| Abnormal pregnancy history | |  |  |  |  |  |  |  |
| No | 0.81 (0.68-0.96) | 0.282 | 0.83 (0.72-0.95) | 0.163 | 1.25 (1.07-1.47) | 0.719 | 1.22 (1.07-1.39) | 0.752 |
| Yes | 0.55 (0.28-1.10) |  | 1.23 (0.72-2.10) |  | 1.44 (0.68-3.08) |  | 1.33 (0.79-2.23) |  |
| Family history of diabetes | |  |  |  |  |  |  |  |
| No | 0.77 (0.64-0.92) | 0.418 | 0.82 (0.71-0.95) | 0.346 | 1.18 (1.00-1.40) | 0.313 | 1.22 (1.07-1.40) | 0.463 |
| Yes | 0.93 (0.61-1.41) |  | 0.97 (0.71-1.34) |  | 1.46 (1.00-2.13) |  | 1.07 (0.77-1.47) |  |
| Note: ^a^ *P* -value for the heterogeneity test. Abbreviation: GDM, gestational diabetes mellitus. | | | | | | | | |

| **Supplementary Table 6. The impacts of rs1121980 and its in high LD SNPs (r^2^ > 0.80) on gene expression based on the PhenoScanner** | | | | | | | | | | | |
| --- | --- | --- | --- | --- | --- | --- | --- | --- | --- | --- | --- |
| SNP | Alleles | Proxy rsID | Proxy Alleles | r^2^ | dprime | Tissue | Exp_gene | Beta | SE | P | N |
| rs1121980 | G/A | rs1121980 | G/A | 1 | 1 | Muscle skeletal | FTO | -0.139 | 0.02943 | 3.16E-06 | 491 |
| rs1121980 | G/A | rs1121980 | G/A | 1 | 1 | Whole blood | SLC22A6 | 0.0074 | 0.00183 | 5.17E-05 | 5257 |
| rs1121980 | G/A | rs1121980 | G/A | 1 | 1 | Peripheral blood | TEX9 | NA | NA | 1.15E-05 | 5311 |
| rs1121980 | G/A | rs1121980 | G/A | 1 | 1 | Whole blood | FTO | NA | NA | 2.78E-06 | 31086 |
| rs1121980 | G/A | rs1121980 | G/A | 1 | 1 | Whole blood | ZNF480 | NA | NA | 2.74E-06 | 25262 |
| rs1121980 | G/A | rs1121980 | G/A | 1 | 1 | Whole blood | TUBB4B | NA | NA | 6.35E-05 | 30381 |
| rs1121980 | G/A | rs1121980 | G/A | 1 | 1 | Whole blood | TAF1A | NA | NA | 6.38E-05 | 31046 |
| rs1121980 | G/A | rs1121980 | G/A | 1 | 1 | Whole blood | TMEM170B | NA | NA | 7.08E-05 | 20737 |
| rs1121980 | G/A | rs1121980 | G/A | 1 | 1 | Whole blood | APOBEC3C | NA | NA | 0.000136 | 31300 |
| rs1121980 | G/A | rs1121980 | G/A | 1 | 1 | Whole blood | RDM1 | NA | NA | 0.000264 | 29228 |
| rs1121980 | G/A | rs1121980 | G/A | 1 | 1 | Whole blood | B3GNT7 | NA | NA | 0.000277 | 31300 |
| rs1121980 | G/A | rs1121980 | G/A | 1 | 1 | Whole blood | ZIK1 | NA | NA | 0.000294 | 26225 |
| rs1121980 | G/A | rs1121980 | G/A | 1 | 1 | Whole blood | ZNF571 | NA | NA | 0.000314 | 31086 |
| rs1121980 | G/A | rs1121980 | G/A | 1 | 1 | Whole blood | SLC18B1 | NA | NA | 0.000338 | 25306 |
| rs1121980 | G/A | rs1121980 | G/A | 1 | 1 | Whole blood | ALMS1P | NA | NA | 0.000346 | 9605 |
| rs1121980 | G/A | rs1121980 | G/A | 1 | 1 | Whole blood | PEX13 | NA | NA | 0.000398 | 31260 |
| rs1121980 | G/A | rs1121980 | G/A | 1 | 1 | Whole blood | ARVCF | NA | NA | 0.000421 | 31300 |
| rs1121980 | G/A | rs1121980 | G/A | 1 | 1 | Whole blood | HIST1H1C | NA | NA | 0.000421 | 31300 |
| rs1121980 | G/A | rs1121980 | G/A | 1 | 1 | Whole blood | AC092155.1 | NA | NA | 0.000427 | 4868 |
| rs1121980 | G/A | rs1121980 | G/A | 1 | 1 | Whole blood | ZBTB7A | NA | NA | 0.000496 | 31300 |
| rs1121980 | G/A | rs1121980 | G/A | 1 | 1 | Whole blood | MDFIC | NA | NA | 0.000506 | 31300 |
| rs1121980 | G/A | rs1121980 | G/A | 1 | 1 | Whole blood | NPFF | NA | NA | 0.000557 | 30475 |
| rs1121980 | G/A | rs1121980 | G/A | 1 | 1 | Whole blood | AGBL2 | NA | NA | 0.00059 | 31300 |
| rs1121980 | G/A | rs1121980 | G/A | 1 | 1 | Whole blood | RP11-498E2.8 | NA | NA | 0.000627 | 4677 |
| rs1121980 | G/A | rs1121980 | G/A | 1 | 1 | Whole blood | HLA-DMB | NA | NA | 0.000663 | 13344 |
| rs1121980 | G/A | rs1121980 | G/A | 1 | 1 | Whole blood | SMARCD3 | NA | NA | 0.00067 | 31086 |
| rs1121980 | G/A | rs1121980 | G/A | 1 | 1 | Whole blood | HDGFRP3 | NA | NA | 0.000681 | 31300 |
| rs1121980 | G/A | rs1121980 | G/A | 1 | 1 | Whole blood | HNRNPA1P16 | NA | NA | 0.000705 | 5502 |
| rs1121980 | G/A | rs1121980 | G/A | 1 | 1 | Whole blood | RP11-96H19.1 | NA | NA | 0.000836 | 5502 |
| rs1121980 | G/A | rs1121980 | G/A | 1 | 1 | Whole blood | KB-1027C11.4 | NA | NA | 0.000858 | 5502 |
| rs1121980 | G/A | rs1121980 | G/A | 1 | 1 | Whole blood | NDST2 | NA | NA | 0.000861 | 31086 |
| rs1121980 | G/A | rs1121980 | G/A | 1 | 1 | Whole blood | TXNDC11 | NA | NA | 0.000946 | 14263 |
| rs1121980 | G/A | rs1121980 | G/A | 1 | 1 | Whole blood | SLC2A9 | NA | NA | 0.000952 | 26225 |
| rs1121980 | G/A | rs201940822 | GACC/G | 1 | 1 | Muscle skeletal | FTO | -0.1317 | 0.03755 | 0.0005164 | 361 |
| rs1121980 | G/A | rs201940822 | GACC/G | 1 | 1 | Muscle skeletal | FTO | -0.1363 | 0.02921 | 4.11E-06 | 491 |
| rs1121980 | G/A | rs201940822 | GACC/G | 1 | 1 | Whole blood | SLC22A6 | NA | NA | 3.29E-05 | 5257 |
| rs1121980 | G/A | rs199952722 | G/GT | 1 | 1 | Muscle skeletal | FTO | -0.1317 | 0.03756 | 0.0005184 | 361 |
| rs1121980 | G/A | rs199952722 | G/GT | 1 | 1 | Muscle skeletal | FTO | -0.1366 | 0.02919 | 3.87E-06 | 491 |
| rs1121980 | G/A | rs199952722 | G/GT | 1 | 1 | Whole blood | SLC22A6 | NA | NA | 3.15E-05 | 5257 |
| rs1121980 | G/A | rs57292959 | G/T | 1 | 1 | Muscle skeletal | FTO | NA | NA | 0.0006722 | 361 |
| rs1121980 | G/A | rs57292959 | G/T | 1 | 1 | Muscle skeletal | FTO | -0.1355 | 0.0293 | 4.94E-06 | 491 |
| rs1121980 | G/A | rs57292959 | G/T | 1 | 1 | Whole blood | SLC22A6 | NA | NA | 3.43E-05 | 5257 |
| rs1121980 | G/A | rs9937521 | C/T | 1 | 1 | Muscle skeletal | FTO | -0.1276 | 0.03783 | 0.0008335 | 361 |
| rs1121980 | G/A | rs9937521 | C/T | 1 | 1 | Muscle skeletal | FTO | -0.1408 | 0.02928 | 2.12E-06 | 491 |
| rs1121980 | G/A | rs9937521 | C/T | 1 | 1 | Whole blood | SLC22A6 | 0.0087 | 0.00197 | 1.04E-05 | 5257 |
| rs1121980 | G/A | rs9937521 | C/T | 1 | 1 | Whole blood | RDM1 | -0.0195 | 0.00499 | 9.42E-05 | 5257 |
| rs1121980 | G/A | rs9937521 | C/T | 1 | 1 | Whole blood | FTO | NA | NA | 2.37E-05 | 26942 |
| rs1121980 | G/A | rs28562191 | C/T | 1 | 1 | Muscle skeletal | FTO | -0.1256 | 0.03733 | 0.0008606 | 361 |
| rs1121980 | G/A | rs28562191 | C/T | 1 | 1 | Muscle skeletal | FTO | -0.1367 | 0.02929 | 4.09E-06 | 491 |
| rs1121980 | G/A | rs28562191 | C/T | 1 | 1 | Whole blood | RDM1 | -0.0198 | 0.00504 | 8.79E-05 | 5257 |
| rs1121980 | G/A | rs28562191 | C/T | 1 | 1 | Whole blood | FTO | NA | NA | 5.92E-05 | 26942 |
| rs1121980 | G/A | rs9937053 | G/A | 1 | 1 | Muscle skeletal | FTO | -0.1346 | 0.0296 | 7.10E-06 | 491 |
| rs1121980 | G/A | rs9937053 | G/A | 1 | 1 | Whole blood | SLC22A6 | 0.0075 | 0.00184 | 4.80E-05 | 5257 |
| rs1121980 | G/A | rs9937053 | G/A | 1 | 1 | Whole blood | FTO | NA | NA | 1.68E-06 | 31355 |
| rs1121980 | G/A | rs9937354 | G/A | 1 | 1 | Muscle skeletal | FTO | -0.1366 | 0.02919 | 3.87E-06 | 491 |
| rs1121980 | G/A | rs9937354 | G/A | 1 | 1 | Whole blood | SLC22A6 | 0.0075 | 0.00184 | 4.74E-05 | 5257 |
| rs1121980 | G/A | rs9937354 | G/A | 1 | 1 | Whole blood | FTO | NA | NA | 2.56E-06 | 31355 |
| rs1121980 | G/A | rs9928094 | A/G | 1 | 1 | Muscle skeletal | FTO | -0.1366 | 0.02919 | 3.87E-06 | 491 |
| rs1121980 | G/A | rs9928094 | A/G | 1 | 1 | Whole blood | SLC22A6 | 0.0075 | 0.00184 | 4.76E-05 | 5257 |
| rs1121980 | G/A | rs9928094 | A/G | 1 | 1 | Whole blood | FTO | NA | NA | 1.88E-06 | 31470 |
| rs1121980 | G/A | rs9930397 | T/A | 1 | 1 | Muscle skeletal | FTO | -0.1341 | 0.02917 | 5.71E-06 | 491 |
| rs1121980 | G/A | rs9930397 | T/A | 1 | 1 | Whole blood | SLC22A6 | 0.0075 | 0.00184 | 4.81E-05 | 5257 |
| rs1121980 | G/A | rs9930397 | T/A | 1 | 1 | Whole blood | FTO | NA | NA | 1.87E-06 | 31355 |
| rs1121980 | G/A | rs9940278 | C/T | 1 | 1 | Muscle skeletal | FTO | -0.1342 | 0.02918 | 5.57E-06 | 491 |
| rs1121980 | G/A | rs9940278 | C/T | 1 | 1 | Whole blood | SLC22A6 | 0.0075 | 0.00184 | 4.74E-05 | 5257 |
| rs1121980 | G/A | rs9940278 | C/T | 1 | 1 | Whole blood | FTO | NA | NA | 1.60E-06 | 31355 |
| rs1121980 | G/A | rs9939973 | G/A | 1 | 1 | Muscle skeletal | FTO | -0.1342 | 0.02918 | 5.57E-06 | 491 |
| rs1121980 | G/A | rs9939973 | G/A | 1 | 1 | Whole blood | SLC22A6 | 0.0075 | 0.00184 | 4.73E-05 | 5257 |
| rs1121980 | G/A | rs9939973 | G/A | 1 | 1 | Whole blood | FTO | NA | NA | 1.32E-06 | 31470 |
| rs1121980 | G/A | rs9940646 | C/G | 1 | 1 | Muscle skeletal | FTO | -0.1325 | 0.02922 | 7.54E-06 | 491 |
| rs1121980 | G/A | rs9940646 | C/G | 1 | 1 | Whole blood | SLC22A6 | 0.0075 | 0.00184 | 4.76E-05 | 5257 |
| rs1121980 | G/A | rs9940646 | C/G | 1 | 1 | Whole blood | FTO | NA | NA | 1.67E-06 | 31354 |
| rs1121980 | G/A | rs1421086 | C/A | 1 | 1 | Muscle skeletal | FTO | -0.1343 | 0.02921 | 5.66E-06 | 491 |
| rs1121980 | G/A | rs1421086 | C/A | 1 | 1 | Whole blood | SLC22A6 | 0.0075 | 0.00184 | 4.79E-05 | 5257 |
| rs1121980 | G/A | rs1421086 | C/A | 1 | 1 | Whole blood | FTO | NA | NA | 1.78E-06 | 31355 |
| rs1121980 | G/A | rs9923544 | C/T | 1 | 1 | Muscle skeletal | FTO | -0.1324 | 0.02925 | 7.83E-06 | 491 |
| rs1121980 | G/A | rs9923544 | C/T | 1 | 1 | Whole blood | SLC22A6 | 0.0075 | 0.00185 | 4.90E-05 | 5257 |
| rs1121980 | G/A | rs9923544 | C/T | 1 | 1 | Whole blood | FTO | NA | NA | 2.02E-06 | 31355 |
| rs1121980 | G/A | rs1558901 | A/T | 1 | 1 | Muscle skeletal | FTO | -0.1325 | 0.02922 | 7.54E-06 | 491 |
| rs1121980 | G/A | rs1558901 | A/T | 1 | 1 | Whole blood | SLC22A6 | 0.0075 | 0.00184 | 4.83E-05 | 5257 |
| rs1121980 | G/A | rs1558901 | A/T | 1 | 1 | Whole blood | FTO | NA | NA | 1.65E-06 | 31354 |
| rs1121980 | G/A | rs11075985 | C/A | 1 | 1 | Muscle skeletal | FTO | -0.1386 | 0.02947 | 3.46E-06 | 491 |
| rs1121980 | G/A | rs11075985 | C/A | 1 | 1 | Whole blood | SLC22A6 | 0.0075 | 0.00184 | 4.86E-05 | 5257 |
| rs1121980 | G/A | rs11075985 | C/A | 1 | 1 | Whole blood | FTO | NA | NA | 3.41E-06 | 31354 |
| rs1121980 | G/A | rs9940128 | G/A | 0.98821 | 1 | Muscle skeletal | FTO | -0.1319 | 0.02925 | 8.44E-06 | 491 |
| rs1121980 | G/A | rs9940128 | G/A | 0.98821 | 1 | Whole blood | SLC22A6 | 0.0075 | 0.00184 | 4.80E-05 | 5257 |
| rs1121980 | G/A | rs9940128 | G/A | 0.98821 | 1 | Whole blood | FTO | NA | NA | 1.28E-06 | 31354 |
| rs1121980 | G/A | rs9940128 | G/A | 0.98821 | 1 | Whole blood | ZNF480 | NA | NA | 3.90E-06 | 25531 |
| rs1121980 | G/A | rs9940128 | G/A | 0.98821 | 1 | Whole blood | TAF1A | NA | NA | 1.56E-05 | 31315 |
| rs1121980 | G/A | rs9940128 | G/A | 0.98821 | 1 | Whole blood | TUBB4B | NA | NA | 3.78E-05 | 30650 |
| rs1121980 | G/A | rs9940128 | G/A | 0.98821 | 1 | Whole blood | TMEM170B | NA | NA | 7.56E-05 | 21006 |
| rs1121980 | G/A | rs9940128 | G/A | 0.98821 | 1 | Whole blood | RDM1 | NA | NA | 9.21E-05 | 29497 |
| rs1121980 | G/A | rs9940128 | G/A | 0.98821 | 1 | Whole blood | APOBEC3C | NA | NA | 0.000112 | 31569 |
| rs1121980 | G/A | rs9940128 | G/A | 0.98821 | 1 | Whole blood | ARVCF | NA | NA | 0.000234 | 31569 |
| rs1121980 | G/A | rs9940128 | G/A | 0.98821 | 1 | Whole blood | SMARCD3 | NA | NA | 0.000293 | 31355 |
| rs1121980 | G/A | rs9940128 | G/A | 0.98821 | 1 | Whole blood | MDFIC | NA | NA | 0.000335 | 31569 |
| rs1121980 | G/A | rs9940128 | G/A | 0.98821 | 1 | Whole blood | ZIK1 | NA | NA | 0.000393 | 26494 |
| rs1121980 | G/A | rs9940128 | G/A | 0.98821 | 1 | Whole blood | ZNF571 | NA | NA | 0.000407 | 31355 |
| rs1121980 | G/A | rs9940128 | G/A | 0.98821 | 1 | Whole blood | ZBTB7A | NA | NA | 0.000409 | 31569 |
| rs1121980 | G/A | rs9940128 | G/A | 0.98821 | 1 | Whole blood | PANK4 | NA | NA | 0.000417 | 31569 |
| rs1121980 | G/A | rs9940128 | G/A | 0.98821 | 1 | Whole blood | HNRNPA1P16 | NA | NA | 0.000419 | 5502 |
| rs1121980 | G/A | rs9940128 | G/A | 0.98821 | 1 | Whole blood | HDGFRP3 | NA | NA | 0.00043 | 31569 |
| rs1121980 | G/A | rs9940128 | G/A | 0.98821 | 1 | Whole blood | SLC18B1 | NA | NA | 0.000472 | 25575 |
| rs1121980 | G/A | rs9940128 | G/A | 0.98821 | 1 | Whole blood | PEX13 | NA | NA | 0.000483 | 31529 |
| rs1121980 | G/A | rs9940128 | G/A | 0.98821 | 1 | Whole blood | AC092155.1 | NA | NA | 0.00049 | 4868 |
| rs1121980 | G/A | rs9940128 | G/A | 0.98821 | 1 | Whole blood | TXNDC11 | NA | NA | 0.000562 | 14263 |
| rs1121980 | G/A | rs9940128 | G/A | 0.98821 | 1 | Whole blood | HLA-DMB | NA | NA | 0.000573 | 13344 |
| rs1121980 | G/A | rs9940128 | G/A | 0.98821 | 1 | Whole blood | KB-1027C11.4 | NA | NA | 0.000597 | 5502 |
| rs1121980 | G/A | rs9940128 | G/A | 0.98821 | 1 | Whole blood | NDST2 | NA | NA | 0.000746 | 31355 |
| rs1121980 | G/A | rs9940128 | G/A | 0.98821 | 1 | Whole blood | ALMS1P | NA | NA | 0.000768 | 9605 |
| rs1121980 | G/A | rs9940128 | G/A | 0.98821 | 1 | Whole blood | RP11-96H19.1 | NA | NA | 0.000781 | 5502 |
| rs1121980 | G/A | rs9940128 | G/A | 0.98821 | 1 | Whole blood | AGBL2 | NA | NA | 0.000817 | 31569 |
| rs1121980 | G/A | rs9940128 | G/A | 0.98821 | 1 | Whole blood | HLCS | NA | NA | 0.000837 | 31529 |
| rs1121980 | G/A | rs9940128 | G/A | 0.98821 | 1 | Whole blood | B3GNT7 | NA | NA | 0.000851 | 31569 |
| rs1121980 | G/A | rs9940128 | G/A | 0.98821 | 1 | Whole blood | MFN2 | NA | NA | 0.000907 | 31569 |
| rs1121980 | G/A | rs9940128 | G/A | 0.98821 | 1 | Whole blood | ARNTL2 | NA | NA | 0.000912 | 30650 |
| rs1121980 | G/A | rs9940128 | G/A | 0.98821 | 1 | Whole blood | ZBTB4 | NA | NA | 0.000923 | 31355 |
| rs1121980 | G/A | rs9940128 | G/A | 0.98821 | 1 | Whole blood | VPS4A | NA | NA | 0.000931 | 31569 |
| rs1121980 | G/A | rs9923147 | C/T | 0.98821 | 1 | Muscle skeletal | FTO | -0.1349 | 0.02965 | 7.06E-06 | 491 |
| rs1121980 | G/A | rs9923147 | C/T | 0.98821 | 1 | Whole blood | SLC22A6 | 0.0075 | 0.00184 | 4.80E-05 | 5257 |
| rs1121980 | G/A | rs9923147 | C/T | 0.98821 | 1 | Whole blood | FTO | NA | NA | 1.91E-06 | 31354 |
| rs1121980 | G/A | rs9933509 | T/C | 0.98821 | 1 | Muscle skeletal | FTO | -0.1256 | 0.02931 | 2.26E-05 | 491 |
| rs1121980 | G/A | rs9933509 | T/C | 0.98821 | 1 | Whole blood | ANKRD11 | -0.0072 | 0.00181 | 7.35E-05 | 5257 |
| rs1121980 | G/A | rs9933509 | T/C | 0.98821 | 1 | Whole blood | FTO | NA | NA | 1.09E-05 | 31354 |
| rs1121980 | G/A | rs9933509 | T/C | 0.98821 | 1 | Whole blood | RBL2 | NA | NA | 0.000958 | 31568 |
| rs1121980 | G/A | rs9931900 | A/G | 0.98821 | 1 | Muscle skeletal | FTO | -0.1254 | 0.02931 | 2.32E-05 | 491 |
| rs1121980 | G/A | rs9931900 | A/G | 0.98821 | 1 | Whole blood | ANKRD11 | -0.0073 | 0.00183 | 6.87E-05 | 5257 |
| rs1121980 | G/A | rs9931900 | A/G | 0.98821 | 1 | Whole blood | FTO | NA | NA | 9.64E-06 | 31355 |
| rs1121980 | G/A | rs9931900 | A/G | 0.98821 | 1 | Whole blood | RBL2 | NA | NA | 0.000998 | 31569 |
| rs1121980 | G/A | rs9937709 | A/G | 0.98821 | 1 | Whole blood | ANKRD11 | -0.0072 | 0.00182 | 7.74E-05 | 5257 |
| rs1121980 | G/A | rs9937709 | A/G | 0.98821 | 1 | Whole blood | FTO | NA | NA | 1.07E-05 | 31355 |
| rs1121980 | G/A | rs7206629 | T/C | 0.98821 | 1 | Muscle skeletal | FTO | -0.125 | 0.02931 | 2.47E-05 | 491 |
| rs1121980 | G/A | rs7206629 | T/C | 0.98821 | 1 | Whole blood | ANKRD11 | -0.0072 | 0.00182 | 7.59E-05 | 5257 |
| rs1121980 | G/A | rs7206629 | T/C | 0.98821 | 1 | Whole blood | FTO | NA | NA | 1.16E-05 | 31355 |
| rs1121980 | G/A | rs7201850 | C/T | 0.98226 | 0.99404 | Muscle skeletal | FTO | -0.1266 | 0.02931 | 1.93E-05 | 491 |
| rs1121980 | G/A | rs7201850 | C/T | 0.98226 | 0.99404 | Whole blood | ANKRD11 | -0.0072 | 0.00182 | 7.47E-05 | 5257 |
| rs1121980 | G/A | rs7201850 | C/T | 0.98226 | 0.99404 | Whole blood | FTO | NA | NA | 9.36E-06 | 31470 |
| rs1121980 | G/A | rs9941349 | C/T | 0.97042 | 0.98804 | Muscle skeletal | FTO | -0.1214 | 0.03022 | 6.94E-05 | 491 |
| rs1121980 | G/A | rs9941349 | C/T | 0.97042 | 0.98804 | Whole blood | FTO | NA | NA | 2.64E-05 | 31085 |
| rs1121980 | G/A | rs9941349 | C/T | 0.97042 | 0.98804 | Whole blood | ZNF480 | NA | NA | 1.47E-05 | 25262 |
| rs1121980 | G/A | rs9941349 | C/T | 0.97042 | 0.98804 | Whole blood | RP11-96H19.1 | NA | NA | 2.00E-05 | 5502 |
| rs1121980 | G/A | rs9941349 | C/T | 0.97042 | 0.98804 | Whole blood | TUBB4B | NA | NA | 7.51E-05 | 30381 |
| rs1121980 | G/A | rs9941349 | C/T | 0.97042 | 0.98804 | Whole blood | ZBTB7A | NA | NA | 0.000165 | 31300 |
| rs1121980 | G/A | rs9941349 | C/T | 0.97042 | 0.98804 | Whole blood | TMEM170B | NA | NA | 0.00021 | 20737 |
| rs1121980 | G/A | rs9941349 | C/T | 0.97042 | 0.98804 | Whole blood | RGPD1 | NA | NA | 0.000264 | 5502 |
| rs1121980 | G/A | rs9941349 | C/T | 0.97042 | 0.98804 | Whole blood | SLC18B1 | NA | NA | 0.000267 | 25306 |
| rs1121980 | G/A | rs9941349 | C/T | 0.97042 | 0.98804 | Whole blood | B3GNT7 | NA | NA | 0.00034 | 31300 |
| rs1121980 | G/A | rs9941349 | C/T | 0.97042 | 0.98804 | Whole blood | SMARCD3 | NA | NA | 0.000343 | 31086 |
| rs1121980 | G/A | rs9941349 | C/T | 0.97042 | 0.98804 | Whole blood | HDGFRP3 | NA | NA | 0.000356 | 31300 |
| rs1121980 | G/A | rs9941349 | C/T | 0.97042 | 0.98804 | Whole blood | TAF1A | NA | NA | 0.000367 | 31046 |
| rs1121980 | G/A | rs9941349 | C/T | 0.97042 | 0.98804 | Whole blood | RP11-498E2.8 | NA | NA | 0.000368 | 4677 |
| rs1121980 | G/A | rs9941349 | C/T | 0.97042 | 0.98804 | Whole blood | PEX13 | NA | NA | 0.000399 | 31260 |
| rs1121980 | G/A | rs9941349 | C/T | 0.97042 | 0.98804 | Whole blood | HORMAD1 | NA | NA | 0.000404 | 31260 |
| rs1121980 | G/A | rs9941349 | C/T | 0.97042 | 0.98804 | Whole blood | MDFIC | NA | NA | 0.000471 | 31300 |
| rs1121980 | G/A | rs9941349 | C/T | 0.97042 | 0.98804 | Whole blood | TAF1C | NA | NA | 0.00048 | 31086 |
| rs1121980 | G/A | rs9941349 | C/T | 0.97042 | 0.98804 | Whole blood | ARNTL2 | NA | NA | 0.000495 | 30381 |
| rs1121980 | G/A | rs9941349 | C/T | 0.97042 | 0.98804 | Whole blood | RDM1 | NA | NA | 0.000498 | 29228 |
| rs1121980 | G/A | rs9941349 | C/T | 0.97042 | 0.98804 | Whole blood | ZNF571 | NA | NA | 0.000508 | 31086 |
| rs1121980 | G/A | rs9941349 | C/T | 0.97042 | 0.98804 | Whole blood | DUSP6 | NA | NA | 0.000537 | 31300 |
| rs1121980 | G/A | rs9941349 | C/T | 0.97042 | 0.98804 | Whole blood | ZIK1 | NA | NA | 0.000559 | 26225 |
| rs1121980 | G/A | rs9941349 | C/T | 0.97042 | 0.98804 | Whole blood | KB-1027C11.4 | NA | NA | 0.000583 | 5502 |
| rs1121980 | G/A | rs9941349 | C/T | 0.97042 | 0.98804 | Whole blood | NDST2 | NA | NA | 0.000629 | 31086 |
| rs1121980 | G/A | rs9941349 | C/T | 0.97042 | 0.98804 | Whole blood | AC092155.1 | NA | NA | 0.000735 | 4868 |
| rs1121980 | G/A | rs9941349 | C/T | 0.97042 | 0.98804 | Whole blood | SAG | NA | NA | 0.000754 | 29218 |
| rs1121980 | G/A | rs9941349 | C/T | 0.97042 | 0.98804 | Whole blood | RGPD2 | NA | NA | 0.000783 | 5502 |
| rs1121980 | G/A | rs9941349 | C/T | 0.97042 | 0.98804 | Whole blood | RP11-335O4.3 | NA | NA | 0.000833 | 3831 |
| rs1121980 | G/A | rs9941349 | C/T | 0.97042 | 0.98804 | Whole blood | PABPC1L | NA | NA | 0.000852 | 20563 |
| rs1121980 | G/A | rs9941349 | C/T | 0.97042 | 0.98804 | Whole blood | NPFF | NA | NA | 0.00089 | 30475 |
| rs1121980 | G/A | rs9941349 | C/T | 0.97042 | 0.98804 | Whole blood | APOBEC3C | NA | NA | 0.000916 | 31300 |
| rs1121980 | G/A | rs9941349 | C/T | 0.97042 | 0.98804 | Whole blood | VPS4A | NA | NA | 0.000927 | 31300 |
| rs1121980 | G/A | rs9941349 | C/T | 0.97042 | 0.98804 | Whole blood | UBE2T | NA | NA | 0.000965 | 31300 |
| rs1121980 | G/A | rs28567725 | T/C | 0.97042 | 0.98804 | Muscle skeletal | FTO | -0.1206 | 0.03001 | 6.95E-05 | 491 |
| rs1121980 | G/A | rs28567725 | T/C | 0.97042 | 0.98804 | Whole blood | FTO | NA | NA | 1.35E-05 | 31354 |
| rs1121980 | G/A | rs28567725 | T/C | 0.97042 | 0.98804 | Whole blood | RBL2 | NA | NA | 0.000671 | 31568 |
| rs1121980 | G/A | rs9931494 | C/G | 0.97042 | 0.98804 | Muscle skeletal | FTO | -0.1224 | 0.03002 | 5.44E-05 | 491 |
| rs1121980 | G/A | rs9931494 | C/G | 0.97042 | 0.98804 | Whole blood | FTO | NA | NA | 2.49E-05 | 31354 |
| rs1121980 | G/A | rs9931494 | C/G | 0.97042 | 0.98804 | Whole blood | RBL2 | NA | NA | 0.000521 | 31568 |
| rs1121980 | G/A | rs9930501 | A/G | 0.92539 | 0.98198 | Muscle skeletal | FTO | -0.1238 | 0.02988 | 4.15E-05 | 491 |
| rs1121980 | G/A | rs9930501 | A/G | 0.92539 | 0.98198 | Whole blood | RDM1 | -0.0182 | 0.00462 | 8.14E-05 | 5257 |
| rs1121980 | G/A | rs9930501 | A/G | 0.92539 | 0.98198 | Whole blood | FTO | NA | NA | 5.54E-05 | 31384 |
| rs1121980 | G/A | rs9930506 | A/G | 0.92539 | 0.98198 | Muscle skeletal | FTO | -0.1238 | 0.03008 | 4.66E-05 | 491 |
| rs1121980 | G/A | rs9930506 | A/G | 0.92539 | 0.98198 | Whole blood | RDM1 | -0.0182 | 0.00462 | 8.16E-05 | 5257 |
| rs1121980 | G/A | rs9930506 | A/G | 0.92539 | 0.98198 | Whole blood | FTO | NA | NA | 8.84E-05 | 30970 |
| rs1121980 | G/A | rs9930506 | A/G | 0.92539 | 0.98198 | Whole blood | ZBTB7A | NA | NA | 7.00E-05 | 31185 |
| rs1121980 | G/A | rs9930506 | A/G | 0.92539 | 0.98198 | Whole blood | TUBB4B | NA | NA | 7.07E-05 | 30266 |
| rs1121980 | G/A | rs9930506 | A/G | 0.92539 | 0.98198 | Whole blood | ZNF480 | NA | NA | 8.50E-05 | 25147 |
| rs1121980 | G/A | rs9930506 | A/G | 0.92539 | 0.98198 | Whole blood | RP11-96H19.1 | NA | NA | 0.00014 | 5502 |
| rs1121980 | G/A | rs9930506 | A/G | 0.92539 | 0.98198 | Whole blood | PEX13 | NA | NA | 0.000148 | 31145 |
| rs1121980 | G/A | rs9930506 | A/G | 0.92539 | 0.98198 | Whole blood | SLC18B1 | NA | NA | 0.000171 | 25191 |
| rs1121980 | G/A | rs9930506 | A/G | 0.92539 | 0.98198 | Whole blood | SAG | NA | NA | 0.000207 | 29103 |
| rs1121980 | G/A | rs9930506 | A/G | 0.92539 | 0.98198 | Whole blood | B3GNT7 | NA | NA | 0.000253 | 31185 |
| rs1121980 | G/A | rs9930506 | A/G | 0.92539 | 0.98198 | Whole blood | MDFIC | NA | NA | 0.000287 | 31185 |
| rs1121980 | G/A | rs9930506 | A/G | 0.92539 | 0.98198 | Whole blood | DUSP6 | NA | NA | 0.000336 | 31185 |
| rs1121980 | G/A | rs9930506 | A/G | 0.92539 | 0.98198 | Whole blood | RDM1 | NA | NA | 0.000364 | 29113 |
| rs1121980 | G/A | rs9930506 | A/G | 0.92539 | 0.98198 | Whole blood | KB-1027C11.4 | NA | NA | 0.000372 | 5502 |
| rs1121980 | G/A | rs9930506 | A/G | 0.92539 | 0.98198 | Whole blood | SSH2 | NA | NA | 0.000428 | 14263 |
| rs1121980 | G/A | rs9930506 | A/G | 0.92539 | 0.98198 | Whole blood | FGF9 | NA | NA | 0.000487 | 31185 |
| rs1121980 | G/A | rs9930506 | A/G | 0.92539 | 0.98198 | Whole blood | HORMAD1 | NA | NA | 0.00053 | 31145 |
| rs1121980 | G/A | rs9930506 | A/G | 0.92539 | 0.98198 | Whole blood | HDGFRP3 | NA | NA | 0.000628 | 31185 |
| rs1121980 | G/A | rs9930506 | A/G | 0.92539 | 0.98198 | Whole blood | AC092155.1 | NA | NA | 0.000673 | 4868 |
| rs1121980 | G/A | rs9930506 | A/G | 0.92539 | 0.98198 | Whole blood | ZIK1 | NA | NA | 0.000727 | 26110 |
| rs1121980 | G/A | rs9930506 | A/G | 0.92539 | 0.98198 | Whole blood | AGBL2 | NA | NA | 0.000816 | 31185 |
| rs1121980 | G/A | rs9930506 | A/G | 0.92539 | 0.98198 | Whole blood | RGPD1 | NA | NA | 0.000868 | 5502 |
| rs1121980 | G/A | rs9930506 | A/G | 0.92539 | 0.98198 | Whole blood | PABPC1L | NA | NA | 0.000882 | 20563 |
| rs1121980 | G/A | rs9930506 | A/G | 0.92539 | 0.98198 | Whole blood | TAF1A | NA | NA | 0.000891 | 30931 |
| rs1121980 | G/A | rs9930506 | A/G | 0.92539 | 0.98198 | Whole blood | UBE2T | NA | NA | 0.000894 | 31185 |
| rs1121980 | G/A | rs9930506 | A/G | 0.92539 | 0.98198 | Whole blood | HNRNPA1P16 | NA | NA | 0.00091 | 5502 |
| rs1121980 | G/A | rs9930506 | A/G | 0.92539 | 0.98198 | Whole blood | RP11-317N8.4 | NA | NA | 0.000916 | 20948 |
| rs1121980 | G/A | rs9930506 | A/G | 0.92539 | 0.98198 | Whole blood | KIDINS220 | NA | NA | 0.000972 | 9188 |
| rs1121980 | G/A | rs9932754 | T/C | 0.92539 | 0.98198 | Muscle skeletal | FTO | -0.1236 | 0.02988 | 4.23E-05 | 491 |
| rs1121980 | G/A | rs9932754 | T/C | 0.92539 | 0.98198 | Whole blood | RDM1 | -0.0182 | 0.00462 | 8.17E-05 | 5257 |
| rs1121980 | G/A | rs9932754 | T/C | 0.92539 | 0.98198 | Whole blood | FTO | NA | NA | 6.35E-05 | 31354 |
| rs1121980 | G/A | rs9933040 | T/A | 0.92539 | 0.98198 | Muscle skeletal | FTO | -0.1238 | 0.02988 | 4.15E-05 | 491 |
| rs1121980 | G/A | rs9933040 | T/A | 0.92539 | 0.98198 | Whole blood | RDM1 | -0.0184 | 0.00468 | 8.46E-05 | 5257 |
| rs1121980 | G/A | rs9933040 | T/A | 0.92539 | 0.98198 | Whole blood | FTO | NA | NA | 5.20E-05 | 31355 |
| rs1121980 | G/A | rs9922708 | C/T | 0.92539 | 0.98198 | Muscle skeletal | FTO | -0.1235 | 0.02993 | 4.44E-05 | 491 |
| rs1121980 | G/A | rs9922708 | C/T | 0.92539 | 0.98198 | Whole blood | SUGT1 | -0.0171 | 0.00429 | 6.87E-05 | 5257 |
| rs1121980 | G/A | rs9922708 | C/T | 0.92539 | 0.98198 | Whole blood | FTO | NA | NA | 5.35E-05 | 31470 |
| rs1121980 | G/A | rs9922619 | G/T | 0.92539 | 0.98198 | Muscle skeletal | FTO | -0.1241 | 0.03038 | 5.31E-05 | 491 |
| rs1121980 | G/A | rs9922619 | G/T | 0.92539 | 0.98198 | Whole blood | RDM1 | -0.0187 | 0.00471 | 7.31E-05 | 5257 |
| rs1121980 | G/A | rs9922619 | G/T | 0.92539 | 0.98198 | Whole blood | FTO | NA | NA | 9.80E-05 | 31470 |

| **Supplementary Table 7. The impacts of rs163182 and its in high LD SNPs (r^2^ > 0.80) on gene expression based on the PhenoScanner** | | | | | | | | | | | |
| --- | --- | --- | --- | --- | --- | --- | --- | --- | --- | --- | --- |
| SNP | Alleles | Proxy rsID | Proxy Alleles | r^2^ | dprime | Tissue | Exp_gene | Beta | SE | P | N |
| rs163182 | C/G | rs163182 | C/G | 1 | 1 | Whole blood | SLC22A18 | NA | NA | 1.37E-08 | 2116 |
| rs163182 | C/G | rs163182 | C/G | 1 | 1 | Whole blood | SLC22A18;  SLC22A18AS | NA | NA | 6.04E-05 | 2116 |
| rs163182 | C/G | rs163182 | C/G | 1 | 1 | Artery aorta | SLC22A18 | -0.3327 | 0.08104 | 6.42E-05 | 197 |
| rs163182 | C/G | rs163182 | C/G | 1 | 1 | Liver | OSBPL5 | 0.3506 | 0.09663 | 0.0005182 | 97 |
| rs163182 | C/G | rs163182 | C/G | 1 | 1 | Artery aorta | SLC22A18 | -0.2651 | 0.06689 | 0.0001008 | 267 |
| rs163182 | C/G | rs163182 | C/G | 1 | 1 | Brain cortex | C11orf21 | 0.4058 | 0.1169 | 0.0007329 | 136 |
| rs163182 | C/G | rs163182 | C/G | 1 | 1 | Brain frontal cortex BA9 | TNNI2 | 0.3938 | 0.115 | 0.0009111 | 118 |
| rs163182 | C/G | rs163182 | C/G | 1 | 1 | Skin not sun exposed suprapubic | C11orf89 | -0.1609 | 0.04676 | 0.000667 | 335 |
| rs163182 | C/G | rs163182 | C/G | 1 | 1 | Whole blood | SLC22A18 | NA | NA | 6.20E-09 | 23651 |
| rs163182 | C/G | rs163182 | C/G | 1 | 1 | Whole blood | CDKN1C | NA | NA | 7.61E-08 | 14252 |
| rs163182 | C/G | rs163182 | C/G | 1 | 1 | Whole blood | SLC22A18AS | NA | NA | 3.54E-07 | 22902 |
| rs163182 | C/G | rs163182 | C/G | 1 | 1 | Whole blood | MINOS1P3 | NA | NA | 0.000127 | 4530 |
| rs163182 | C/G | rs163182 | C/G | 1 | 1 | Whole blood | TET3 | NA | NA | 0.000131 | 14263 |
| rs163182 | C/G | rs163182 | C/G | 1 | 1 | Whole blood | ZNF197 | NA | NA | 0.000132 | 23448 |
| rs163182 | C/G | rs163182 | C/G | 1 | 1 | Whole blood | MRPL44 | NA | NA | 0.000177 | 23622 |
| rs163182 | C/G | rs163182 | C/G | 1 | 1 | Whole blood | TOR1B | NA | NA | 0.000314 | 23662 |
| rs163182 | C/G | rs163182 | C/G | 1 | 1 | Whole blood | THYN1 | NA | NA | 0.000316 | 23662 |
| rs163182 | C/G | rs163182 | C/G | 1 | 1 | Whole blood | DPPA4 | NA | NA | 0.000374 | 23662 |
| rs163182 | C/G | rs163182 | C/G | 1 | 1 | Whole blood | USP36 | NA | NA | 0.000376 | 23448 |
| rs163182 | C/G | rs163182 | C/G | 1 | 1 | Whole blood | ZFAND2A | NA | NA | 0.000473 | 23662 |
| rs163182 | C/G | rs163182 | C/G | 1 | 1 | Whole blood | SEPN1 | NA | NA | 0.000542 | 23662 |
| rs163182 | C/G | rs163182 | C/G | 1 | 1 | Whole blood | UCHL5 | NA | NA | 0.000543 | 22913 |
| rs163182 | C/G | rs163182 | C/G | 1 | 1 | Whole blood | EDEM1 | NA | NA | 0.00056 | 23448 |
| rs163182 | C/G | rs163182 | C/G | 1 | 1 | Whole blood | MGAT4B | NA | NA | 0.000613 | 23662 |
| rs163182 | C/G | rs163182 | C/G | 1 | 1 | Whole blood | AGMAT | NA | NA | 0.00064 | 23662 |
| rs163182 | C/G | rs163182 | C/G | 1 | 1 | Whole blood | ACMSD | NA | NA | 0.000822 | 21410 |
| rs163182 | C/G | rs163182 | C/G | 1 | 1 | Whole blood | TMEM208 | NA | NA | 0.000864 | 19126 |
| rs163182 | C/G | rs163182 | C/G | 1 | 1 | Whole blood | NRAS | NA | NA | 0.000939 | 23662 |
| rs163182 | C/G | rs163182 | C/G | 1 | 1 | Whole blood | RP1-283E3.8 | NA | NA | 0.000939 | 12620 |
| rs163182 | C/G | rs163182 | C/G | 1 | 1 | Whole blood | PBX4 | NA | NA | 0.000943 | 23662 |
| rs163182 | C/G | rs163182 | C/G | 1 | 1 | Whole blood | OCLM | NA | NA | 0.000996 | 22235 |

| **Supplementary Table 8. The impacts of rs12970134 and its in high LD SNPs (r^2^ > 0.80) on gene expression based on the PhenoScanner** | | | | | | | | | | | |
| --- | --- | --- | --- | --- | --- | --- | --- | --- | --- | --- | --- |
| SNP | Alleles | Proxy rsID | Proxy Alleles | r^2^ | dprime | Tissue | Exp_gene | Beta | SE | P | N |
| rs12970134 | A/G | rs12970134 | A/G | 1 | 1 | Blood | CTNNA1 | NA | NA | 8.80E-06 | 1469 |
| rs12970134 | A/G | rs12970134 | A/G | 1 | 1 | Lung | PMAIP1 | 0.1545 | 0.04405 | 0.0005192 | 383 |
| rs12970134 | A/G | rs12970134 | A/G | 1 | 1 | Whole blood | NLGN2 | 0.0131 | 0.00262 | 5.81E-07 | 5257 |
| rs12970134 | A/G | rs12970134 | A/G | 1 | 1 | Whole blood | OR8H1;OR8I1P | 0.0379 | 0.0084 | 6.56E-06 | 5257 |
| rs12970134 | A/G | rs12970134 | A/G | 1 | 1 | Whole blood | ACSBG1;IDH3A | -0.0109 | 0.00255 | 2.00E-05 | 5257 |
| rs12970134 | A/G | rs12970134 | A/G | 1 | 1 | Whole blood | FBXW4P1 | NA | NA | 0.000111 | 13438 |
| rs12970134 | A/G | rs12970134 | A/G | 1 | 1 | Whole blood | COL4A1 | NA | NA | 0.000145 | 31300 |
| rs12970134 | A/G | rs12970134 | A/G | 1 | 1 | Whole blood | AC009501.4 | NA | NA | 0.000206 | 18311 |
| rs12970134 | A/G | rs12970134 | A/G | 1 | 1 | Whole blood | RWDD4P1 | NA | NA | 0.000263 | 3831 |
| rs12970134 | A/G | rs12970134 | A/G | 1 | 1 | Whole blood | TTC19 | NA | NA | 0.000265 | 31086 |
| rs12970134 | A/G | rs12970134 | A/G | 1 | 1 | Whole blood | U6 | NA | NA | 0.000506 | 3831 |
| rs12970134 | A/G | rs12970134 | A/G | 1 | 1 | Whole blood | RP11-214O1.2 | NA | NA | 0.000619 | 5502 |
| rs12970134 | A/G | rs12970134 | A/G | 1 | 1 | Whole blood | LINC00334 | NA | NA | 0.000629 | 17973 |
| rs12970134 | A/G | rs12970134 | A/G | 1 | 1 | Whole blood | PROCA1 | NA | NA | 0.000671 | 31300 |
| rs12970134 | A/G | rs12970134 | A/G | 1 | 1 | Whole blood | Y_RNA | NA | NA | 0.000693 | 3831 |
| rs12970134 | A/G | rs12970134 | A/G | 1 | 1 | Whole blood | CTB-58E17.1 | NA | NA | 0.000713 | 22111 |
| rs12970134 | A/G | rs12970134 | A/G | 1 | 1 | Whole blood | RSPRY1 | NA | NA | 0.00076 | 31300 |
| rs12970134 | A/G | rs12970134 | A/G | 1 | 1 | Whole blood | B3GAT1 | NA | NA | 0.000809 | 31300 |
| rs12970134 | A/G | rs12970134 | A/G | 1 | 1 | Whole blood | ECHDC1 | NA | NA | 0.000883 | 31086 |
| rs12970134 | A/G | rs12970134 | A/G | 1 | 1 | Whole blood | IGKV3-7 | NA | NA | 0.000885 | 4677 |
| rs12970134 | A/G | rs12970134 | A/G | 1 | 1 | Whole blood | MRPL20 | NA | NA | 0.000938 | 26011 |
| rs12970134 | A/G | rs12970134 | A/G | 1 | 1 | Whole blood | RP11-1149O23.3 | NA | NA | 0.000959 | 22111 |
| rs12970134 | A/G | rs2045439 | T/C | 1 | 1 | Lung | PMAIP1 | 0.1539 | 0.04397 | 0.0005298 | 383 |
| rs12970134 | A/G | rs2045439 | T/C | 1 | 1 | Whole blood | NLGN2 | 0.0131 | 0.00262 | 5.83E-07 | 5257 |
| rs12970134 | A/G | rs2045439 | T/C | 1 | 1 | Whole blood | OR8H1;OR8I1P | 0.0379 | 0.0084 | 6.50E-06 | 5257 |
| rs12970134 | A/G | rs2045439 | T/C | 1 | 1 | Whole blood | ACSBG1;IDH3A | -0.011 | 0.00257 | 1.96E-05 | 5257 |
| rs12970134 | A/G | rs12957325 | C/T | 1 | 1 | Whole blood | NLGN2 | 0.0131 | 0.00263 | 6.22E-07 | 5257 |
| rs12970134 | A/G | rs12957325 | C/T | 1 | 1 | Whole blood | OR8H1;OR8I1P | 0.0383 | 0.00838 | 5.03E-06 | 5257 |
| rs12970134 | A/G | rs12957325 | C/T | 1 | 1 | Whole blood | ACSBG1;IDH3A | -0.0109 | 0.00255 | 2.01E-05 | 5257 |
| rs12970134 | A/G | rs11660069 | G/A | 1 | 1 | Lung | PMAIP1 | 0.1539 | 0.04397 | 0.0005298 | 383 |
| rs12970134 | A/G | rs11660069 | G/A | 1 | 1 | Whole blood | NLGN2 | 0.0132 | 0.00264 | 5.80E-07 | 5257 |
| rs12970134 | A/G | rs11660069 | G/A | 1 | 1 | Whole blood | OR8H1;OR8I1P | 0.0379 | 0.0084 | 6.55E-06 | 5257 |
| rs12970134 | A/G | rs11660069 | G/A | 1 | 1 | Whole blood | ACSBG1;IDH3A | -0.0109 | 0.00256 | 2.05E-05 | 5257 |
| rs12970134 | A/G | rs35476226 | G/C | 1 | 1 | Lung | PMAIP1 | 0.1539 | 0.04397 | 0.0005298 | 383 |
| rs12970134 | A/G | rs35476226 | G/C | 1 | 1 | Whole blood | NLGN2 | 0.0132 | 0.00264 | 5.80E-07 | 5257 |
| rs12970134 | A/G | rs35476226 | G/C | 1 | 1 | Whole blood | OR8H1;OR8I1P | 0.0379 | 0.0084 | 6.55E-06 | 5257 |
| rs12970134 | A/G | rs35476226 | G/C | 1 | 1 | Whole blood | ACSBG1;IDH3A | -0.0109 | 0.00256 | 2.07E-05 | 5257 |
| rs12970134 | A/G | rs12955983 | G/A | 0.99309 | 1 | Whole blood | NLGN2 | 0.0131 | 0.00263 | 6.41E-07 | 5257 |
| rs12970134 | A/G | rs12955983 | G/A | 0.99309 | 1 | Whole blood | OR8H1;OR8I1P | 0.0387 | 0.00839 | 4.04E-06 | 5257 |
| rs12970134 | A/G | rs12955983 | G/A | 0.99309 | 1 | Whole blood | ACSBG1;IDH3A | -0.011 | 0.00256 | 1.72E-05 | 5257 |
| rs12970134 | A/G | rs2045438 | T/C | 0.99309 | 1 | Lung | PMAIP1 | 0.1529 | 0.04401 | 0.0005864 | 383 |
| rs12970134 | A/G | rs2045438 | T/C | 0.99309 | 1 | Whole blood | NLGN2 | 0.0131 | 0.00263 | 6.32E-07 | 5257 |
| rs12970134 | A/G | rs2045438 | T/C | 0.99309 | 1 | Whole blood | OR8H1;OR8I1P | 0.0387 | 0.00838 | 3.94E-06 | 5257 |
| rs12970134 | A/G | rs2045438 | T/C | 0.99309 | 1 | Whole blood | ACSBG1;IDH3A | -0.0108 | 0.00255 | 2.34E-05 | 5257 |
| rs12970134 | A/G | rs67713315 | G/A | 0.98627 | 1 | Whole blood | NLGN2 | 0.0131 | 0.00263 | 6.31E-07 | 5257 |
| rs12970134 | A/G | rs67713315 | G/A | 0.98627 | 1 | Whole blood | OR8H1;OR8I1P | 0.0384 | 0.00841 | 5.06E-06 | 5257 |
| rs12970134 | A/G | rs67713315 | G/A | 0.98627 | 1 | Whole blood | ACSBG1;IDH3A | -0.0107 | 0.00257 | 3.23E-05 | 5257 |
| rs12970134 | A/G | rs11665439 | G/A | 0.98627 | 1 | Whole blood | NLGN2 | 0.0135 | 0.00268 | 4.86E-07 | 5257 |
| rs12970134 | A/G | rs11665439 | G/A | 0.98627 | 1 | Whole blood | OR8H1;OR8I1P | 0.0389 | 0.00855 | 5.45E-06 | 5257 |
| rs12970134 | A/G | rs11665439 | G/A | 0.98627 | 1 | Whole blood | ACSBG1;IDH3A | -0.0109 | 0.00261 | 3.05E-05 | 5257 |
| rs12970134 | A/G | rs11661876 | A/G | 0.98627 | 1 | Whole blood | NLGN2 | 0.0136 | 0.00269 | 4.60E-07 | 5257 |
| rs12970134 | A/G | rs11661876 | A/G | 0.98627 | 1 | Whole blood | OR8H1;OR8I1P | 0.0393 | 0.0086 | 4.97E-06 | 5257 |
| rs12970134 | A/G | rs11661876 | A/G | 0.98627 | 1 | Whole blood | ACSBG1;IDH3A | -0.011 | 0.00262 | 2.80E-05 | 5257 |
| rs12970134 | A/G | rs67194783 | T/C | 0.98627 | 1 | Whole blood | NLGN2 | 0.0131 | 0.00263 | 6.60E-07 | 5257 |
| rs12970134 | A/G | rs67194783 | T/C | 0.98627 | 1 | Whole blood | OR8H1;OR8I1P | 0.0385 | 0.00842 | 4.88E-06 | 5257 |
| rs12970134 | A/G | rs67194783 | T/C | 0.98627 | 1 | Whole blood | ACSBG1;IDH3A | -0.0107 | 0.00259 | 3.61E-05 | 5257 |
| rs12970134 | A/G | rs11665563 | T/C | 0.94055 | 1 | Whole blood | NLGN2 | 0.0131 | 0.00264 | 6.86E-07 | 5257 |
| rs12970134 | A/G | rs11665563 | T/C | 0.94055 | 1 | Whole blood | OR8H1;OR8I1P | 0.0386 | 0.0084 | 4.37E-06 | 5257 |
| rs12970134 | A/G | rs11665563 | T/C | 0.94055 | 1 | Whole blood | ACSBG1;IDH3A | -0.011 | 0.00255 | 1.66E-05 | 5257 |
| rs12970134 | A/G | rs11663816 | C/T | 0.94055 | 1 | Whole blood | NLGN2 | 0.0129 | 0.00262 | 8.41E-07 | 5257 |
| rs12970134 | A/G | rs11663816 | C/T | 0.94055 | 1 | Whole blood | OR8H1;OR8I1P | 0.0386 | 0.00839 | 4.33E-06 | 5257 |
| rs12970134 | A/G | rs11663816 | C/T | 0.94055 | 1 | Whole blood | ACSBG1;IDH3A | -0.011 | 0.00257 | 1.84E-05 | 5257 |
| rs12970134 | A/G | rs11664883 | A/T | 0.94055 | 1 | Whole blood | NLGN2 | 0.0131 | 0.00263 | 6.77E-07 | 5257 |
| rs12970134 | A/G | rs11664883 | A/T | 0.94055 | 1 | Whole blood | OR8H1;OR8I1P | 0.0385 | 0.00838 | 4.49E-06 | 5257 |
| rs12970134 | A/G | rs11664883 | A/T | 0.94055 | 1 | Whole blood | ACSBG1;IDH3A | -0.011 | 0.00256 | 1.71E-05 | 5257 |
| rs12970134 | A/G | rs17175602 | C/T | 0.94055 | 1 | Lung | PMAIP1 | 0.1539 | 0.04397 | 0.0005298 | 383 |
| rs12970134 | A/G | rs17175602 | C/T | 0.94055 | 1 | Whole blood | NLGN2 | 0.0131 | 0.00262 | 6.02E-07 | 5257 |
| rs12970134 | A/G | rs17175602 | C/T | 0.94055 | 1 | Whole blood | OR8H1;OR8I1P | 0.0379 | 0.00838 | 6.30E-06 | 5257 |
| rs12970134 | A/G | rs17175602 | C/T | 0.94055 | 1 | Whole blood | ACSBG1;IDH3A | -0.011 | 0.00256 | 1.80E-05 | 5257 |
| rs12970134 | A/G | rs12964203 | C/T | 0.94055 | 1 | Whole blood | NLGN2 | 0.0131 | 0.00265 | 7.83E-07 | 5257 |
| rs12970134 | A/G | rs12964203 | C/T | 0.94055 | 1 | Whole blood | OR8H1;OR8I1P | 0.0386 | 0.00844 | 4.89E-06 | 5257 |
| rs12970134 | A/G | rs12964203 | C/T | 0.94055 | 1 | Whole blood | ACSBG1;IDH3A | -0.0106 | 0.00258 | 3.94E-05 | 5257 |
| rs12970134 | A/G | rs590215 | T/C | 0.94055 | 1 | Whole blood | NLGN2 | 0.0129 | 0.00265 | 1.12E-06 | 5257 |
| rs12970134 | A/G | rs590215 | T/C | 0.94055 | 1 | Whole blood | OR8H1;OR8I1P | 0.038 | 0.00845 | 7.08E-06 | 5257 |
| rs12970134 | A/G | rs590215 | T/C | 0.94055 | 1 | Whole blood | ACSBG1;IDH3A | -0.0107 | 0.00259 | 3.69E-05 | 5257 |
| rs12970134 | A/G | rs656384 | A/G | 0.94055 | 1 | Whole blood | NLGN2 | 0.0131 | 0.00264 | 7.34E-07 | 5257 |
| rs12970134 | A/G | rs656384 | A/G | 0.94055 | 1 | Whole blood | OR8H1;OR8I1P | 0.037 | 0.00848 | 1.30E-05 | 5257 |
| rs12970134 | A/G | rs656384 | A/G | 0.94055 | 1 | Whole blood | ACSBG1;IDH3A | -0.0103 | 0.0026 | 7.32E-05 | 5257 |
| rs12970134 | A/G | rs2168708 | T/G | 0.94055 | 1 | Whole blood | NLGN2 | 0.0131 | 0.00265 | 8.28E-07 | 5257 |
| rs12970134 | A/G | rs2168708 | T/G | 0.94055 | 1 | Whole blood | OR8H1;OR8I1P | 0.0387 | 0.00845 | 4.80E-06 | 5257 |
| rs12970134 | A/G | rs2168708 | T/G | 0.94055 | 1 | Whole blood | ACSBG1;IDH3A | -0.0107 | 0.00259 | 3.75E-05 | 5257 |
| rs12970134 | A/G | rs11665052 | G/A | 0.94055 | 1 | Whole blood | NLGN2 | 0.0131 | 0.00266 | 8.80E-07 | 5257 |
| rs12970134 | A/G | rs11665052 | G/A | 0.94055 | 1 | Whole blood | OR8H1;OR8I1P | 0.0386 | 0.00848 | 5.38E-06 | 5257 |
| rs12970134 | A/G | rs11665052 | G/A | 0.94055 | 1 | Whole blood | ACSBG1;IDH3A | -0.0108 | 0.00259 | 3.09E-05 | 5257 |
| rs12970134 | A/G | rs12966550 | G/A | 0.94055 | 1 | Whole blood | NLGN2 | 0.013 | 0.00265 | 9.40E-07 | 5257 |
| rs12970134 | A/G | rs12966550 | G/A | 0.94055 | 1 | Whole blood | OR8H1;OR8I1P | 0.0391 | 0.00848 | 4.11E-06 | 5257 |
| rs12970134 | A/G | rs12966550 | G/A | 0.94055 | 1 | Whole blood | ACSBG1;IDH3A | -0.0108 | 0.00259 | 3.00E-05 | 5257 |
| rs12970134 | A/G | rs146693753 | A/AAC | 0.94055 | 1 | Whole blood | NLGN2 | NA | NA | 8.98E-07 | 5257 |
| rs12970134 | A/G | rs146693753 | A/AAC | 0.94055 | 1 | Whole blood | OR8H1;OR8I1P | NA | NA | 4.15E-06 | 5257 |
| rs12970134 | A/G | rs146693753 | A/AAC | 0.94055 | 1 | Whole blood | ACSBG1;IDH3A | NA | NA | 2.93E-05 | 5257 |
| rs12970134 | A/G | rs56804022 | C/T | 0.93432 | 1 | Whole blood | NLGN2 | 0.0131 | 0.00263 | 6.31E-07 | 5257 |
| rs12970134 | A/G | rs56804022 | C/T | 0.93432 | 1 | Whole blood | OR8H1;OR8I1P | 0.0378 | 0.0084 | 7.02E-06 | 5257 |
| rs12970134 | A/G | rs56804022 | C/T | 0.93432 | 1 | Whole blood | ACSBG1;IDH3A | -0.0106 | 0.00257 | 3.70E-05 | 5257 |
| rs12970134 | A/G | rs12956871 | G/A | 0.93432 | 1 | Whole blood | NLGN2 | 0.0131 | 0.00263 | 6.46E-07 | 5257 |
| rs12970134 | A/G | rs12956871 | G/A | 0.93432 | 1 | Whole blood | OR8H1;OR8I1P | 0.0378 | 0.0084 | 6.88E-06 | 5257 |
| rs12970134 | A/G | rs12956871 | G/A | 0.93432 | 1 | Whole blood | ACSBG1;IDH3A | -0.0105 | 0.00257 | 4.45E-05 | 5257 |
| rs12970134 | A/G | rs12960928 | C/T | 0.93432 | 1 | Whole blood | NLGN2 | 0.0131 | 0.00263 | 6.47E-07 | 5257 |
| rs12970134 | A/G | rs12960928 | C/T | 0.93432 | 1 | Whole blood | OR8H1;OR8I1P | 0.0378 | 0.0084 | 6.89E-06 | 5257 |
| rs12970134 | A/G | rs12960928 | C/T | 0.93432 | 1 | Whole blood | ACSBG1;IDH3A | -0.0105 | 0.00257 | 4.47E-05 | 5257 |
| rs12970134 | A/G | rs34893777 | T/A | 0.93432 | 1 | Whole blood | NLGN2 | 0.0131 | 0.00265 | 8.01E-07 | 5257 |
| rs12970134 | A/G | rs34893777 | T/A | 0.93432 | 1 | Whole blood | OR8H1;OR8I1P | 0.0386 | 0.00844 | 4.86E-06 | 5257 |
| rs12970134 | A/G | rs34893777 | T/A | 0.93432 | 1 | Whole blood | ACSBG1;IDH3A | -0.0106 | 0.00257 | 3.85E-05 | 5257 |
| rs12970134 | A/G | rs17175713 | C/T | 0.93432 | 1 | Lung | PMAIP1 | 0.1507 | 0.04397 | 0.0006889 | 383 |
| rs12970134 | A/G | rs17175713 | C/T | 0.93432 | 1 | Whole blood | NLGN2 | 0.0133 | 0.00268 | 7.40E-07 | 5257 |
| rs12970134 | A/G | rs17175713 | C/T | 0.93432 | 1 | Whole blood | OR8H1;OR8I1P | 0.0395 | 0.00857 | 4.14E-06 | 5257 |
| rs12970134 | A/G | rs17175713 | C/T | 0.93432 | 1 | Whole blood | ACSBG1;IDH3A | -0.0106 | 0.00262 | 5.16E-05 | 5257 |
| rs12970134 | A/G | rs17175643 | T/C | 0.92814 | 1 | Whole blood | NLGN2 | 0.0132 | 0.00263 | 5.58E-07 | 5257 |
| rs12970134 | A/G | rs17175643 | T/C | 0.92814 | 1 | Whole blood | OR8H1;OR8I1P | 0.0382 | 0.0084 | 5.51E-06 | 5257 |
| rs12970134 | A/G | rs17175643 | T/C | 0.92814 | 1 | Whole blood | ACSBG1;IDH3A | -0.0107 | 0.00257 | 3.22E-05 | 5257 |
| rs12970134 | A/G | rs35555097 | C/CT | 0.92814 | 1 | Whole blood | NLGN2 | NA | NA | 1.61E-06 | 5257 |
| rs12970134 | A/G | rs35555097 | C/CT | 0.92814 | 1 | Whole blood | OR8H1;OR8I1P | NA | NA | 3.03E-05 | 5257 |
| rs12970134 | A/G | rs8089364 | C/T | 0.90309 | 0.99294 | Lung | PMAIP1 | 0.1482 | 0.04402 | 0.0008553 | 383 |
| rs12970134 | A/G | rs8089364 | C/T | 0.90309 | 0.99294 | Whole blood | NLGN2 | 0.0133 | 0.0027 | 8.80E-07 | 5257 |
| rs12970134 | A/G | rs8089364 | C/T | 0.90309 | 0.99294 | Whole blood | OR8H1;OR8I1P | 0.0406 | 0.00863 | 2.58E-06 | 5257 |
| rs12970134 | A/G | rs8089364 | C/T | 0.90309 | 0.99294 | Whole blood | ACSBG1;IDH3A | -0.0108 | 0.00264 | 4.35E-05 | 5257 |
| rs12970134 | A/G | rs8089364 | C/T | 0.90309 | 0.99294 | Whole blood | RWDD4P1 | NA | NA | 0.000149 | 3831 |
| rs12970134 | A/G | rs8089364 | C/T | 0.90309 | 0.99294 | Whole blood | FBXW4P1 | NA | NA | 0.000191 | 13438 |
| rs12970134 | A/G | rs8089364 | C/T | 0.90309 | 0.99294 | Whole blood | COL4A1 | NA | NA | 0.000348 | 31569 |
| rs12970134 | A/G | rs8089364 | C/T | 0.90309 | 0.99294 | Whole blood | U6 | NA | NA | 0.000495 | 3831 |
| rs12970134 | A/G | rs8089364 | C/T | 0.90309 | 0.99294 | Whole blood | CCNK | NA | NA | 0.000509 | 31569 |
| rs12970134 | A/G | rs8089364 | C/T | 0.90309 | 0.99294 | Whole blood | AC009501.4 | NA | NA | 0.000591 | 18695 |
| rs12970134 | A/G | rs8089364 | C/T | 0.90309 | 0.99294 | Whole blood | PROCA1 | NA | NA | 0.000615 | 31569 |
| rs12970134 | A/G | rs8089364 | C/T | 0.90309 | 0.99294 | Whole blood | TTC19 | NA | NA | 0.000698 | 31355 |
| rs12970134 | A/G | rs8089364 | C/T | 0.90309 | 0.99294 | Whole blood | ECHDC1 | NA | NA | 0.000732 | 30971 |
| rs12970134 | A/G | rs8089364 | C/T | 0.90309 | 0.99294 | Whole blood | LINC00334 | NA | NA | 0.000758 | 18357 |
| rs12970134 | A/G | rs8089364 | C/T | 0.90309 | 0.99294 | Whole blood | CCDC58 | NA | NA | 8.00E-04 | 6421 |
| rs12970134 | A/G | rs8089364 | C/T | 0.90309 | 0.99294 | Whole blood | ITPR1-AS1 | NA | NA | 0.000859 | 4530 |
| rs12970134 | A/G | rs8089364 | C/T | 0.90309 | 0.99294 | Whole blood | IGKV3-7 | NA | NA | 0.000865 | 4677 |
| rs12970134 | A/G | rs8089364 | C/T | 0.90309 | 0.99294 | Whole blood | B3GAT1 | NA | NA | 0.000935 | 31569 |
| rs12970134 | A/G | rs8089364 | C/T | 0.90309 | 0.99294 | Whole blood | RSPRY1 | NA | NA | 0.000952 | 31569 |
| rs12970134 | A/G | rs8089364 | C/T | 0.90309 | 0.99294 | Whole blood | SNX10 | NA | NA | 0.000984 | 31355 |
| rs12970134 | A/G | rs8089364 | C/T | 0.90309 | 0.99294 | Whole blood | B3GNT1 | NA | NA | 0.000991 | 25546 |
| rs12970134 | A/G | rs12969709 | A/C | 0.90309 | 0.99294 | Lung | PMAIP1 | 0.1479 | 0.0439 | 0.0008512 | 383 |
| rs12970134 | A/G | rs12969709 | A/C | 0.90309 | 0.99294 | Whole blood | NLGN2 | 0.0136 | 0.00272 | 5.78E-07 | 5257 |
| rs12970134 | A/G | rs12969709 | A/C | 0.90309 | 0.99294 | Whole blood | OR8H1;OR8I1P | 0.0401 | 0.00867 | 3.87E-06 | 5257 |
| rs12970134 | A/G | rs12969709 | A/C | 0.90309 | 0.99294 | Whole blood | ACSBG1;IDH3A | -0.0109 | 0.00265 | 4.05E-05 | 5257 |
| rs12970134 | A/G | rs921971 | C/T | 0.90309 | 0.99294 | Lung | PMAIP1 | 0.1482 | 0.04402 | 0.0008553 | 383 |
| rs12970134 | A/G | rs921971 | C/T | 0.90309 | 0.99294 | Whole blood | NLGN2 | 0.0133 | 0.0027 | 8.45E-07 | 5257 |
| rs12970134 | A/G | rs921971 | C/T | 0.90309 | 0.99294 | Whole blood | OR8H1;OR8I1P | 0.0406 | 0.00862 | 2.58E-06 | 5257 |
| rs12970134 | A/G | rs921971 | C/T | 0.90309 | 0.99294 | Whole blood | ACSBG1;IDH3A | -0.0108 | 0.00264 | 4.30E-05 | 5257 |
| rs12970134 | A/G | rs74805059 | T/TTC | 0.90309 | 0.99294 | Lung | PMAIP1 | 0.1482 | 0.04402 | 0.0008553 | 383 |
| rs12970134 | A/G | rs74805059 | T/TTC | 0.90309 | 0.99294 | Whole blood | NLGN2 | NA | NA | 8.37E-07 | 5257 |
| rs12970134 | A/G | rs74805059 | T/TTC | 0.90309 | 0.99294 | Whole blood | OR8H1;OR8I1P | NA | NA | 2.58E-06 | 5257 |
| rs12970134 | A/G | rs74805059 | T/TTC | 0.90309 | 0.99294 | Whole blood | ACSBG1;IDH3A | NA | NA | 4.31E-05 | 5257 |
| rs12970134 | A/G | rs12958167 | G/T | 0.90309 | 0.99294 | Whole blood | NLGN2 | 0.0133 | 0.00264 | 4.98E-07 | 5257 |
| rs12970134 | A/G | rs12958167 | G/T | 0.90309 | 0.99294 | Whole blood | OR8H1;OR8I1P | 0.0394 | 0.00843 | 3.05E-06 | 5257 |
| rs12970134 | A/G | rs12958167 | G/T | 0.90309 | 0.99294 | Whole blood | ACSBG1;IDH3A | -0.0108 | 0.00256 | 2.57E-05 | 5257 |
| rs12970134 | A/G | rs34712774 | CG/C | 0.90309 | 0.99294 | Whole blood | NLGN2 | NA | NA | 4.61E-07 | 5257 |
| rs12970134 | A/G | rs34712774 | CG/C | 0.90309 | 0.99294 | Whole blood | OR8H1;OR8I1P | NA | NA | 3.01E-06 | 5257 |
| rs12970134 | A/G | rs34712774 | CG/C | 0.90309 | 0.99294 | Whole blood | ACSBG1;IDH3A | NA | NA | 2.75E-05 | 5257 |
| rs12970134 | A/G | rs8097210 | G/T | 0.89719 | 0.99294 | Lung | PMAIP1 | 0.1482 | 0.04402 | 0.0008553 | 383 |
| rs12970134 | A/G | rs8097210 | G/T | 0.89719 | 0.99294 | Whole blood | NLGN2 | 0.0133 | 0.0027 | 9.02E-07 | 5257 |
| rs12970134 | A/G | rs8097210 | G/T | 0.89719 | 0.99294 | Whole blood | OR8H1;OR8I1P | 0.0406 | 0.00863 | 2.59E-06 | 5257 |
| rs12970134 | A/G | rs8097210 | G/T | 0.89719 | 0.99294 | Whole blood | ACSBG1;IDH3A | -0.0108 | 0.00264 | 4.36E-05 | 5257 |
| rs12970134 | A/G | rs185575165 | A/T | 0.89719 | 0.99294 | Whole blood | THOC1;USP14 | 0.0278 | 0.00711 | 9.37E-05 | 5257 |
| rs12970134 | A/G | rs11354399 | T/TG | 0.87077 | 0.97181 | Whole blood | NLGN2 | NA | NA | 9.99E-07 | 5257 |
| rs12970134 | A/G | rs11354399 | T/TG | 0.87077 | 0.97181 | Whole blood | OR8H1;OR8I1P | NA | NA | 2.48E-06 | 5257 |
| rs12970134 | A/G | rs11354399 | T/TG | 0.87077 | 0.97181 | Whole blood | ACSBG1;IDH3A | NA | NA | 4.49E-05 | 5257 |
| rs12970134 | A/G | rs9955666 | A/G | 0.86434 | 1 | Thyroid | CCBE1 | -0.178 | 0.04423 | 7.05E-05 | 399 |
| rs12970134 | A/G | rs9955666 | A/G | 0.86434 | 1 | Whole blood | NLGN2 | 0.0137 | 0.00247 | 3.26E-08 | 5257 |
| rs12970134 | A/G | rs9955666 | A/G | 0.86434 | 1 | Whole blood | OR8H1;OR8I1P | 0.0329 | 0.00789 | 3.11E-05 | 5257 |
| rs12970134 | A/G | rs9955666 | A/G | 0.86434 | 1 | Whole blood | FAM8A1;FAM8A3P;FAM8A4P;FAM8A5P;FAM8A6P | 0.0086 | 0.00214 | 5.98E-05 | 5257 |
| rs12970134 | A/G | rs1942872 | T/C | 0.86299 | 0.99288 | Lung | PMAIP1 | 0.1482 | 0.04402 | 0.0008553 | 383 |
| rs12970134 | A/G | rs1942872 | T/C | 0.86299 | 0.99288 | Whole blood | NLGN2 | 0.0132 | 0.00263 | 5.38E-07 | 5257 |
| rs12970134 | A/G | rs1942872 | T/C | 0.86299 | 0.99288 | Whole blood | OR8H1;OR8I1P | 0.0388 | 0.00841 | 4.06E-06 | 5257 |
| rs12970134 | A/G | rs1942872 | T/C | 0.86299 | 0.99288 | Whole blood | ACSBG1;IDH3A | -0.0108 | 0.00256 | 2.58E-05 | 5257 |
| rs12970134 | A/G | rs12954782 | G/C | 0.85749 | 0.99287 | Whole blood | NLGN2 | 0.0133 | 0.00265 | 5.17E-07 | 5257 |
| rs12970134 | A/G | rs12954782 | G/C | 0.85749 | 0.99287 | Whole blood | OR8H1;OR8I1P | 0.0392 | 0.00841 | 3.25E-06 | 5257 |
| rs12970134 | A/G | rs12954782 | G/C | 0.85749 | 0.99287 | Whole blood | ACSBG1;IDH3A | -0.0108 | 0.00256 | 2.56E-05 | 5257 |
| rs12970134 | A/G | rs68033110 | A/G | 0.81854 | 0.93407 | Esophagus muscularis | MC4R | 0.2719 | 0.07989 | 0.0008174 | 218 |
| rs12970134 | A/G | rs68033110 | A/G | 0.81854 | 0.93407 | Whole blood | NLGN2 | 0.013 | 0.00298 | 1.28E-05 | 5257 |
| rs12970134 | A/G | rs68033110 | A/G | 0.81854 | 0.93407 | Whole blood | ACSBG1;IDH3A | -0.0115 | 0.00291 | 7.97E-05 | 5257 |
| rs12970134 | A/G | rs8084515 | T/A | 0.80732 | 1 | Thyroid | CCBE1 | -0.1805 | 0.04403 | 5.19E-05 | 399 |
| rs12970134 | A/G | rs8084515 | T/A | 0.80732 | 1 | Whole blood | NLGN2 | 0.014 | 0.00243 | 8.63E-09 | 5257 |
| rs12970134 | A/G | rs8084515 | T/A | 0.80732 | 1 | Whole blood | FAM8A1;FAM8A3P;FAM8A4P;FAM8A5P;FAM8A6P | 0.0082 | 0.0021 | 9.64E-05 | 5257 |
| rs12970134 | A/G | rs9947403 | T/C | 0.80244 | 1 | Brain cerebellar hemisphere | GRP | 0.3365 | 0.09526 | 0.0006189 | 125 |
| rs12970134 | A/G | rs9947403 | T/C | 0.80244 | 1 | Thyroid | CCBE1 | -0.1754 | 0.04366 | 7.29E-05 | 399 |
| rs12970134 | A/G | rs9947403 | T/C | 0.80244 | 1 | Whole blood | NLGN2 | 0.014 | 0.00244 | 9.50E-09 | 5257 |
| rs12970134 | A/G | rs9947403 | T/C | 0.80244 | 1 | Whole blood | FAM8A1;FAM8A3P;FAM8A4P;FAM8A5P;FAM8A6P | 0.0084 | 0.00211 | 7.06E-05 | 5257 |
| rs12970134 | A/G | rs9947403 | T/C | 0.80244 | 1 | Whole blood | OR8H1;OR8I1P | 0.0308 | 0.00777 | 7.52E-05 | 5257 |
| rs12970134 | A/G | rs11366970 | C/CT | 0.80244 | 1 | Thyroid | CCBE1 | -0.188 | 0.04535 | 4.32E-05 | 399 |
| rs12970134 | A/G | rs528074 | G/T | 0.80244 | 1 | Thyroid | CCBE1 | -0.1715 | 0.04374 | 0.0001069 | 399 |
| rs12970134 | A/G | rs528074 | G/T | 0.80244 | 1 | Whole blood | NLGN2 | 0.0135 | 0.00245 | 3.68E-08 | 5257 |
| rs12970134 | A/G | rs528074 | G/T | 0.80244 | 1 | Whole blood | FAM8A1;FAM8A3P;FAM8A4P;FAM8A5P;FAM8A6P | 0.0082 | 0.0021 | 9.74E-05 | 5257 |
| rs12970134 | A/G | rs553731 | A/G | 0.80244 | 1 | Thyroid | CCBE1 | -0.1682 | 0.04384 | 0.0001491 | 399 |
| rs12970134 | A/G | rs553731 | A/G | 0.80244 | 1 | Whole blood | NLGN2 | 0.0135 | 0.00245 | 3.78E-08 | 5257 |
| rs12970134 | A/G | rs553731 | A/G | 0.80244 | 1 | Whole blood | FAM8A1;FAM8A3P;FAM8A4P;FAM8A5P;FAM8A6P | 0.0084 | 0.00213 | 7.91E-05 | 5257 |
| rs12970134 | A/G | rs473381 | T/G | 0.80244 | 1 | Thyroid | CCBE1 | -0.1682 | 0.04384 | 0.0001491 | 399 |
| rs12970134 | A/G | rs473381 | T/G | 0.80244 | 1 | Whole blood | NLGN2 | 0.0135 | 0.00245 | 3.92E-08 | 5257 |
| rs12970134 | A/G | rs473381 | T/G | 0.80244 | 1 | Whole blood | FAM8A1;FAM8A3P;FAM8A4P;FAM8A5P;FAM8A6P | 0.0083 | 0.0021 | 8.13E-05 | 5257 |

| **Supplementary Table 9. The impacts of rs340841 and its in high LD SNPs (r^2^ > 0.80) on gene expression based on the PhenoScanner** | | | | | | | | | | | |
| --- | --- | --- | --- | --- | --- | --- | --- | --- | --- | --- | --- |
| SNP | Alleles | Proxy rsID | Proxy Alleles | r^2^ | dprime | Tissue | Exp_gene | Beta | SE | *P* | N |
| rs340841 | C/T | rs340841 | C/T | 1 | 1 | Brain cerebellar hemisphere | PROX1-AS1 | -0.3109 | 0.0745 | 8.83E-05 | 89 |
| rs340841 | C/T | rs340841 | C/T | 1 | 1 | Brain cerebellum | PROX1-AS1 | -0.3213 | 0.08298 | 0.0002171 | 103 |
| rs340841 | C/T | rs340841 | C/T | 1 | 1 | Brain hippocampus | PROX1-AS1 | -0.2759 | 0.07503 | 0.0005121 | 81 |
| rs340841 | C/T | rs340841 | C/T | 1 | 1 | Brain cerebellar hemisphere | PROX1-AS1 | -0.3073 | 0.06964 | 2.52E-05 | 125 |
| rs340841 | C/T | rs340841 | C/T | 1 | 1 | Brain cerebellum | PROX1-AS1 | -0.3778 | 0.06787 | 1.68E-07 | 154 |
| rs340841 | C/T | rs340841 | C/T | 1 | 1 | Brain hippocampus | PROX1-AS1 | -0.2824 | 0.07615 | 0.0003627 | 111 |
| rs340841 | C/T | rs340841 | C/T | 1 | 1 | Brain putamen basal ganglia | PROX1-AS1 | -0.2291 | 0.06161 | 0.0003495 | 111 |
| rs340841 | C/T | rs340841 | C/T | 1 | 1 | Whole blood | CC2D2B;LOC100653119;LOC100652732 | -0.0114 | 0.00283 | 5.84E-05 | 5257 |
| rs340841 | C/T | rs340847 | G/A | 0.99602 | 1 | Brain cerebellar hemisphere | PROX1-AS1 | -0.3109 | 0.0745 | 8.83E-05 | 89 |
| rs340841 | C/T | rs340847 | G/A | 0.99602 | 1 | Brain cerebellum | PROX1-AS1 | -0.3214 | 0.08298 | 0.0002171 | 103 |
| rs340841 | C/T | rs340847 | G/A | 0.99602 | 1 | Brain hippocampus | PROX1-AS1 | -0.2759 | 0.07503 | 0.0005121 | 81 |
| rs340841 | C/T | rs340847 | G/A | 0.99602 | 1 | Brain cerebellar hemisphere | PROX1-AS1 | -0.3073 | 0.06964 | 2.52E-05 | 125 |
| rs340841 | C/T | rs340847 | G/A | 0.99602 | 1 | Brain cerebellum | PROX1-AS1 | -0.3778 | 0.06787 | 1.68E-07 | 154 |
| rs340841 | C/T | rs340847 | G/A | 0.99602 | 1 | Brain hippocampus | PROX1-AS1 | -0.2824 | 0.07615 | 0.0003627 | 111 |
| rs340841 | C/T | rs340847 | G/A | 0.99602 | 1 | Brain putamen basal ganglia | PROX1-AS1 | -0.2291 | 0.06161 | 0.0003495 | 111 |
| rs340841 | C/T | rs340847 | G/A | 0.99602 | 1 | Whole blood | CC2D2B;LOC100653119;LOC100652732 | -0.0114 | 0.00284 | 6.02E-05 | 5257 |
| rs340841 | C/T | rs340846 | T/C | 0.99602 | 1 | Brain cerebellar hemisphere | PROX1-AS1 | -0.3109 | 0.0745 | 8.83E-05 | 89 |
| rs340841 | C/T | rs340846 | T/C | 0.99602 | 1 | Brain cerebellum | PROX1-AS1 | -0.3213 | 0.08298 | 0.0002171 | 103 |
| rs340841 | C/T | rs340846 | T/C | 0.99602 | 1 | Brain hippocampus | PROX1-AS1 | -0.2759 | 0.07503 | 0.0005121 | 81 |
| rs340841 | C/T | rs340846 | T/C | 0.99602 | 1 | Brain cerebellar hemisphere | PROX1-AS1 | -0.3073 | 0.06964 | 2.52E-05 | 125 |
| rs340841 | C/T | rs340846 | T/C | 0.99602 | 1 | Brain cerebellum | PROX1-AS1 | -0.3778 | 0.06787 | 1.68E-07 | 154 |
| rs340841 | C/T | rs340846 | T/C | 0.99602 | 1 | Brain hippocampus | PROX1-AS1 | -0.2824 | 0.07615 | 0.0003627 | 111 |
| rs340841 | C/T | rs340846 | T/C | 0.99602 | 1 | Brain putamen basal ganglia | PROX1-AS1 | -0.2291 | 0.06161 | 0.0003495 | 111 |
| rs340841 | C/T | rs340846 | T/C | 0.99602 | 1 | Whole blood | CC2D2B;LOC100653119;LOC100652732 | -0.0114 | 0.00284 | 6.01E-05 | 5257 |
| rs340841 | C/T | rs340849 | C/A | 0.99208 | 1 | Brain cerebellar hemisphere | PROX1-AS1 | -0.3109 | 0.0745 | 8.83E-05 | 89 |
| rs340841 | C/T | rs340849 | C/A | 0.99208 | 1 | Brain cerebellum | PROX1-AS1 | -0.3213 | 0.08298 | 0.0002171 | 103 |
| rs340841 | C/T | rs340849 | C/A | 0.99208 | 1 | Brain hippocampus | PROX1-AS1 | -0.2759 | 0.07503 | 0.0005121 | 81 |
| rs340841 | C/T | rs340849 | C/A | 0.99208 | 1 | Brain cerebellar hemisphere | PROX1-AS1 | -0.3073 | 0.06964 | 2.52E-05 | 125 |
| rs340841 | C/T | rs340849 | C/A | 0.99208 | 1 | Brain cerebellum | PROX1-AS1 | -0.3778 | 0.06787 | 1.68E-07 | 154 |
| rs340841 | C/T | rs340849 | C/A | 0.99208 | 1 | Brain hippocampus | PROX1-AS1 | -0.2824 | 0.07615 | 0.0003627 | 111 |
| rs340841 | C/T | rs340849 | C/A | 0.99208 | 1 | Brain putamen basal ganglia | PROX1-AS1 | -0.2291 | 0.06161 | 0.0003495 | 111 |
| rs340841 | C/T | rs340849 | C/A | 0.99208 | 1 | Whole blood | CC2D2B;LOC100653119;LOC100652732 | -0.0113 | 0.00283 | 6.58E-05 | 5257 |
| rs340841 | C/T | rs1008431 | C/G | 0.98816 | 1 | Brain cerebellar hemisphere | PROX1-AS1 | -0.3107 | 0.07454 | 9.01E-05 | 89 |
| rs340841 | C/T | rs1008431 | C/G | 0.98816 | 1 | Brain cerebellum | PROX1-AS1 | -0.3214 | 0.08302 | 0.0002185 | 103 |
| rs340841 | C/T | rs1008431 | C/G | 0.98816 | 1 | Brain hippocampus | PROX1-AS1 | -0.2758 | 0.07504 | 0.0005137 | 81 |
| rs340841 | C/T | rs1008431 | C/G | 0.98816 | 1 | Brain cerebellar hemisphere | PROX1-AS1 | -0.3073 | 0.06964 | 2.52E-05 | 125 |
| rs340841 | C/T | rs1008431 | C/G | 0.98816 | 1 | Brain cerebellum | PROX1-AS1 | -0.3778 | 0.06787 | 1.68E-07 | 154 |
| rs340841 | C/T | rs1008431 | C/G | 0.98816 | 1 | Brain hippocampus | PROX1-AS1 | -0.2824 | 0.07615 | 0.0003627 | 111 |
| rs340841 | C/T | rs1008431 | C/G | 0.98816 | 1 | Brain putamen basal ganglia | PROX1-AS1 | -0.2291 | 0.06161 | 0.0003495 | 111 |
| rs340841 | C/T | rs1008431 | C/G | 0.98816 | 1 | Pancreas | PROX1-AS1 | -0.2977 | 0.08704 | 0.0007725 | 220 |
| rs340841 | C/T | rs1008431 | C/G | 0.98816 | 1 | Whole blood | CC2D2B;LOC100653119;LOC100652732 | -0.0113 | 0.00284 | 7.13E-05 | 5257 |
| rs340841 | C/T | rs11120231 | C/A | 0.98816 | 1 | Brain cerebellar hemisphere | PROX1-AS1 | -0.3109 | 0.0745 | 8.83E-05 | 89 |
| rs340841 | C/T | rs11120231 | C/A | 0.98816 | 1 | Brain cerebellum | PROX1-AS1 | -0.3213 | 0.08298 | 0.0002171 | 103 |
| rs340841 | C/T | rs11120231 | C/A | 0.98816 | 1 | Brain hippocampus | PROX1-AS1 | -0.2759 | 0.07503 | 0.0005121 | 81 |
| rs340841 | C/T | rs11120231 | C/A | 0.98816 | 1 | Brain cerebellar hemisphere | PROX1-AS1 | -0.3073 | 0.06964 | 2.52E-05 | 125 |
| rs340841 | C/T | rs11120231 | C/A | 0.98816 | 1 | Brain cerebellum | PROX1-AS1 | -0.3778 | 0.06787 | 1.68E-07 | 154 |
| rs340841 | C/T | rs11120231 | C/A | 0.98816 | 1 | Brain hippocampus | PROX1-AS1 | -0.2824 | 0.07615 | 0.0003627 | 111 |
| rs340841 | C/T | rs11120231 | C/A | 0.98816 | 1 | Brain putamen basal ganglia | PROX1-AS1 | -0.2291 | 0.06161 | 0.0003495 | 111 |
| rs340841 | C/T | rs11120231 | C/A | 0.98816 | 1 | Whole blood | CC2D2B;LOC100653119;LOC100652732 | -0.0113 | 0.00284 | 7.21E-05 | 5257 |
| rs340841 | C/T | rs10746444 | C/G | 0.98026 | 0.996 | Brain cerebellar hemisphere | PROX1-AS1 | -0.3006 | 0.0764 | 2.00E-04 | 89 |
| rs340841 | C/T | rs10746444 | C/G | 0.98026 | 0.996 | Brain cerebellum | PROX1-AS1 | -0.3315 | 0.08423 | 0.0001743 | 103 |
| rs340841 | C/T | rs10746444 | C/G | 0.98026 | 0.996 | Brain hippocampus | PROX1-AS1 | -0.2735 | 0.07609 | 0.0006647 | 81 |
| rs340841 | C/T | rs10746444 | C/G | 0.98026 | 0.996 | Brain cerebellar hemisphere | PROX1-AS1 | -0.3073 | 0.06964 | 2.52E-05 | 125 |
| rs340841 | C/T | rs10746444 | C/G | 0.98026 | 0.996 | Brain cerebellum | PROX1-AS1 | -0.3778 | 0.06787 | 1.68E-07 | 154 |
| rs340841 | C/T | rs10746444 | C/G | 0.98026 | 0.996 | Brain hippocampus | PROX1-AS1 | -0.2824 | 0.07615 | 0.0003627 | 111 |
| rs340841 | C/T | rs10746444 | C/G | 0.98026 | 0.996 | Brain putamen basal ganglia | PROX1-AS1 | -0.2291 | 0.06161 | 0.0003495 | 111 |
| rs340841 | C/T | rs10746444 | C/G | 0.98026 | 0.996 | Whole blood | CC2D2B;LOC100653119;LOC100652732 | -0.0113 | 0.00286 | 7.78E-05 | 5257 |
| rs340841 | C/T | rs340864 | G/A | 0.89284 | 0.9754 | Brain cerebellar hemisphere | PROX1-AS1 | -0.2845 | 0.0781 | 0.0005271 | 89 |
| rs340841 | C/T | rs340864 | G/A | 0.89284 | 0.9754 | Brain cerebellum | PROX1-AS1 | -0.2933 | 0.08567 | 0.0009707 | 103 |
| rs340841 | C/T | rs340864 | G/A | 0.89284 | 0.9754 | Brain hippocampus | PROX1-AS1 | -0.2925 | 0.07774 | 0.0003899 | 81 |
| rs340841 | C/T | rs340864 | G/A | 0.89284 | 0.9754 | Brain cerebellum | PROX1-AS1 | -0.3362 | 0.06968 | 4.27E-06 | 154 |
| rs340841 | C/T | rs340864 | G/A | 0.89284 | 0.9754 | Brain putamen basal ganglia | PROX1-AS1 | -0.2383 | 0.06513 | 0.0004277 | 111 |
| rs340841 | C/T | rs340863 | G/A | 0.89284 | 0.9754 | Peripheral blood monocytes | SORD | NA | NA | 1.92E-06 | 1490 |
| rs340841 | C/T | rs340863 | G/A | 0.89284 | 0.9754 | Brain cerebellar hemisphere | PROX1-AS1 | -0.2833 | 0.07877 | 0.0006103 | 89 |
| rs340841 | C/T | rs340863 | G/A | 0.89284 | 0.9754 | Brain cerebellum | PROX1-AS1 | -0.2946 | 0.08578 | 0.0009388 | 103 |
| rs340841 | C/T | rs340863 | G/A | 0.89284 | 0.9754 | Brain hippocampus | PROX1-AS1 | -0.2928 | 0.07799 | 0.000399 | 81 |
| rs340841 | C/T | rs340863 | G/A | 0.89284 | 0.9754 | Brain cerebellum | PROX1-AS1 | -0.3328 | 0.07018 | 6.00E-06 | 154 |
| rs340841 | C/T | rs340863 | G/A | 0.89284 | 0.9754 | Brain putamen basal ganglia | PROX1-AS1 | -0.2383 | 0.06513 | 0.0004277 | 111 |
| rs340841 | C/T | rs443335 | C/A | 0.88921 | 0.97535 | Brain cerebellar hemisphere | PROX1-AS1 | -0.2845 | 0.07885 | 0.0005908 | 89 |
| rs340841 | C/T | rs443335 | C/A | 0.88921 | 0.97535 | Brain cerebellum | PROX1-AS1 | -0.2954 | 0.086 | 0.0009386 | 103 |
| rs340841 | C/T | rs443335 | C/A | 0.88921 | 0.97535 | Brain hippocampus | PROX1-AS1 | -0.2938 | 0.07813 | 0.0003918 | 81 |
| rs340841 | C/T | rs443335 | C/A | 0.88921 | 0.97535 | Brain cerebellum | PROX1-AS1 | -0.3328 | 0.07018 | 6.00E-06 | 154 |
| rs340841 | C/T | rs443335 | C/A | 0.88921 | 0.97535 | Brain putamen basal ganglia | PROX1-AS1 | -0.2383 | 0.06513 | 0.0004277 | 111 |
| rs340841 | C/T | rs340868 | T/A | 0.88898 | 0.97136 | Brain cerebellar hemisphere | PROX1-AS1 | -0.3227 | 0.07484 | 5.44E-05 | 89 |
| rs340841 | C/T | rs340868 | T/A | 0.88898 | 0.97136 | Brain cerebellum | PROX1-AS1 | -0.3211 | 0.08312 | 0.0002243 | 103 |
| rs340841 | C/T | rs340868 | T/A | 0.88898 | 0.97136 | Brain hippocampus | PROX1-AS1 | -0.2963 | 0.0754 | 0.0002259 | 81 |
| rs340841 | C/T | rs340868 | T/A | 0.88898 | 0.97136 | Brain cerebellar hemisphere | PROX1-AS1 | -0.3073 | 0.06964 | 2.52E-05 | 125 |
| rs340841 | C/T | rs340868 | T/A | 0.88898 | 0.97136 | Brain cerebellum | PROX1-AS1 | -0.3778 | 0.06787 | 1.68E-07 | 154 |
| rs340841 | C/T | rs340868 | T/A | 0.88898 | 0.97136 | Brain hippocampus | PROX1-AS1 | -0.2824 | 0.07615 | 0.0003627 | 111 |
| rs340841 | C/T | rs340868 | T/A | 0.88898 | 0.97136 | Brain putamen basal ganglia | PROX1-AS1 | -0.2291 | 0.06161 | 0.0003495 | 111 |
| rs340841 | C/T | rs340868 | T/A | 0.88898 | 0.97136 | Whole blood | CC2D2B;LOC100653119;LOC100652732 | -0.0114 | 0.00284 | 6.20E-05 | 5257 |
| rs340841 | C/T | rs2075425 | G/A | 0.86777 | 0.975 | Peripheral blood monocytes | FADS2 | NA | NA | 8.65E-06 | 1490 |
| rs340841 | C/T | rs2075425 | G/A | 0.86777 | 0.975 | Brain cerebellar hemisphere | PROX1-AS1 | -0.2731 | 0.07508 | 0.0005366 | 89 |
| rs340841 | C/T | rs2075425 | G/A | 0.86777 | 0.975 | Brain cerebellum | PROX1-AS1 | -0.3168 | 0.08683 | 0.0004661 | 103 |
| rs340841 | C/T | rs2075425 | G/A | 0.86777 | 0.975 | Brain hippocampus | PROX1-AS1 | -0.3427 | 0.07662 | 3.58E-05 | 81 |
| rs340841 | C/T | rs2075425 | G/A | 0.86777 | 0.975 | Brain cerebellar hemisphere | PROX1-AS1 | -0.2834 | 0.07119 | 0.0001282 | 125 |
| rs340841 | C/T | rs2075425 | G/A | 0.86777 | 0.975 | Brain cerebellum | PROX1-AS1 | -0.3294 | 0.07204 | 1.21E-05 | 154 |
| rs340841 | C/T | rs2075425 | G/A | 0.86777 | 0.975 | Brain hippocampus | PROX1-AS1 | -0.3484 | 0.07598 | 1.48E-05 | 111 |
| rs340841 | C/T | rs2075425 | G/A | 0.86777 | 0.975 | Brain putamen basal ganglia | PROX1-AS1 | -0.2326 | 0.05893 | 0.0001573 | 111 |
| rs340841 | C/T | rs2075425 | G/A | 0.86777 | 0.975 | Whole blood | NLRP9 | -0.0109 | 0.00257 | 2.30E-05 | 5257 |
| rs340841 | C/T | rs340858 | T/C | 0.83653 | 0.9746 | Brain cerebellar hemisphere | PROX1-AS1 | -0.2941 | 0.07434 | 0.0001866 | 89 |
| rs340841 | C/T | rs340858 | T/C | 0.83653 | 0.9746 | Brain cerebellum | PROX1-AS1 | -0.3007 | 0.0858 | 0.0007483 | 103 |
| rs340841 | C/T | rs340858 | T/C | 0.83653 | 0.9746 | Brain hippocampus | PROX1-AS1 | -0.3496 | 0.07365 | 1.36E-05 | 81 |
| rs340841 | C/T | rs340858 | T/C | 0.83653 | 0.9746 | Brain cerebellar hemisphere | PROX1-AS1 | -0.3044 | 0.07209 | 5.21E-05 | 125 |
| rs340841 | C/T | rs340858 | T/C | 0.83653 | 0.9746 | Brain cerebellum | PROX1-AS1 | -0.3624 | 0.0705 | 1.11E-06 | 154 |
| rs340841 | C/T | rs340858 | T/C | 0.83653 | 0.9746 | Brain hippocampus | PROX1-AS1 | -0.3502 | 0.07663 | 1.57E-05 | 111 |
| rs340841 | C/T | rs340858 | T/C | 0.83653 | 0.9746 | Brain putamen basal ganglia | PROX1-AS1 | -0.2445 | 0.05936 | 8.49E-05 | 111 |
| rs340841 | C/T | rs340858 | T/C | 0.83653 | 0.9746 | Blood | PROX1 | NA | NA | 1.16E-05 | 600 |
| rs340841 | C/T | rs340858 | T/C | 0.83653 | 0.9746 | Skeletal muscle | PROX1 | NA | NA | 1.16E-05 | 600 |
| rs340841 | C/T | rs340867 | G/A | 0.83611 | 0.97049 | Brain cerebellar hemisphere | PROX1-AS1 | -0.2937 | 0.07414 | 0.0001833 | 89 |
| rs340841 | C/T | rs340867 | G/A | 0.83611 | 0.97049 | Brain cerebellum | PROX1-AS1 | -0.2974 | 0.08568 | 0.000833 | 103 |
| rs340841 | C/T | rs340867 | G/A | 0.83611 | 0.97049 | Brain hippocampus | PROX1-AS1 | -0.349 | 0.07356 | 1.37E-05 | 81 |
| rs340841 | C/T | rs340867 | G/A | 0.83611 | 0.97049 | Brain cerebellar hemisphere | PROX1-AS1 | -0.3024 | 0.07212 | 5.82E-05 | 125 |
| rs340841 | C/T | rs340867 | G/A | 0.83611 | 0.97049 | Brain cerebellum | PROX1-AS1 | -0.3515 | 0.07082 | 2.38E-06 | 154 |
| rs340841 | C/T | rs340867 | G/A | 0.83611 | 0.97049 | Brain hippocampus | PROX1-AS1 | -0.351 | 0.0766 | 1.49E-05 | 111 |
| rs340841 | C/T | rs340867 | G/A | 0.83611 | 0.97049 | Brain putamen basal ganglia | PROX1-AS1 | -0.2468 | 0.05917 | 7.06E-05 | 111 |
| rs340841 | C/T | rs340867 | G/A | 0.83611 | 0.97049 | Thyroid | SMYD2 | -0.09399 | 0.02826 | 0.0009808 | 399 |
| rs340841 | C/T | rs340854 | A/G | 0.83313 | 0.97455 | Brain cerebellar hemisphere | PROX1-AS1 | -0.2941 | 0.07434 | 0.0001867 | 89 |
| rs340841 | C/T | rs340854 | A/G | 0.83313 | 0.97455 | Brain cerebellum | PROX1-AS1 | -0.3005 | 0.08573 | 0.0007479 | 103 |
| rs340841 | C/T | rs340854 | A/G | 0.83313 | 0.97455 | Brain hippocampus | PROX1-AS1 | -0.3497 | 0.07364 | 1.35E-05 | 81 |
| rs340841 | C/T | rs340854 | A/G | 0.83313 | 0.97455 | Brain cerebellar hemisphere | PROX1-AS1 | -0.3044 | 0.07209 | 5.21E-05 | 125 |
| rs340841 | C/T | rs340854 | A/G | 0.83313 | 0.97455 | Brain cerebellum | PROX1-AS1 | -0.3595 | 0.07063 | 1.38E-06 | 154 |
| rs340841 | C/T | rs340854 | A/G | 0.83313 | 0.97455 | Brain hippocampus | PROX1-AS1 | -0.3502 | 0.07663 | 1.57E-05 | 111 |
| rs340841 | C/T | rs340854 | A/G | 0.83313 | 0.97455 | Brain putamen basal ganglia | PROX1-AS1 | -0.2445 | 0.05936 | 8.49E-05 | 111 |

**Supplementary Figures 1-5**

**
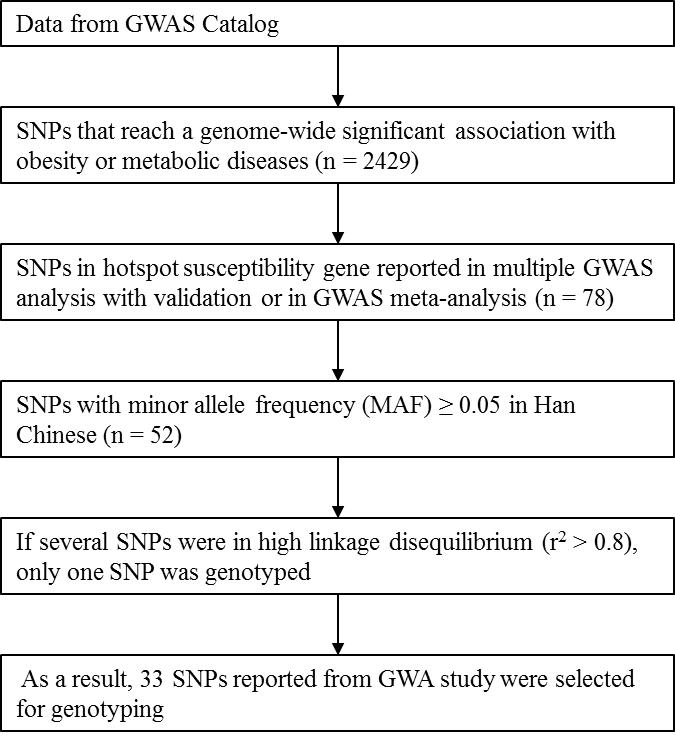
**

**Supplementary Figure 1. Flow chart of the SNP selection**

**
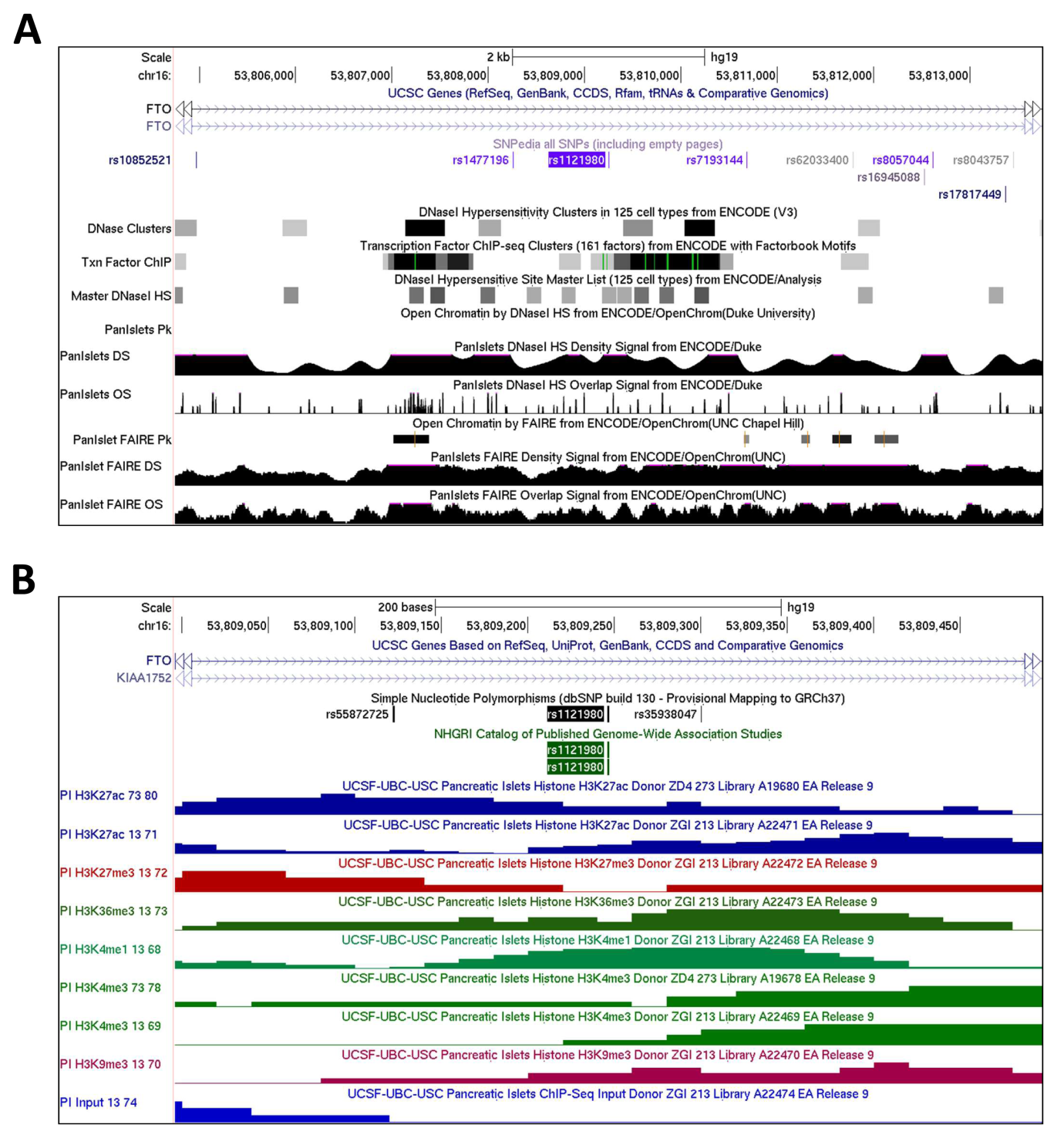
**

**Supplementary Figure 2. Chromatin features of rs1121980.** Functional annotation in proximity to rs1121980 in pancreatic islets from ENCODE (A) and Roadmap data (B). DNaseI hypersensitivity (HS) clusters, the Formaldehyde-Assisted Isolation of Regulatory Elements (FAIRE) density signal, and several histone modification markers (such as H3K27ac, H3K27me3, H3K36me3, H3K4me1, H3K4me3, and H3K9me3) are present.


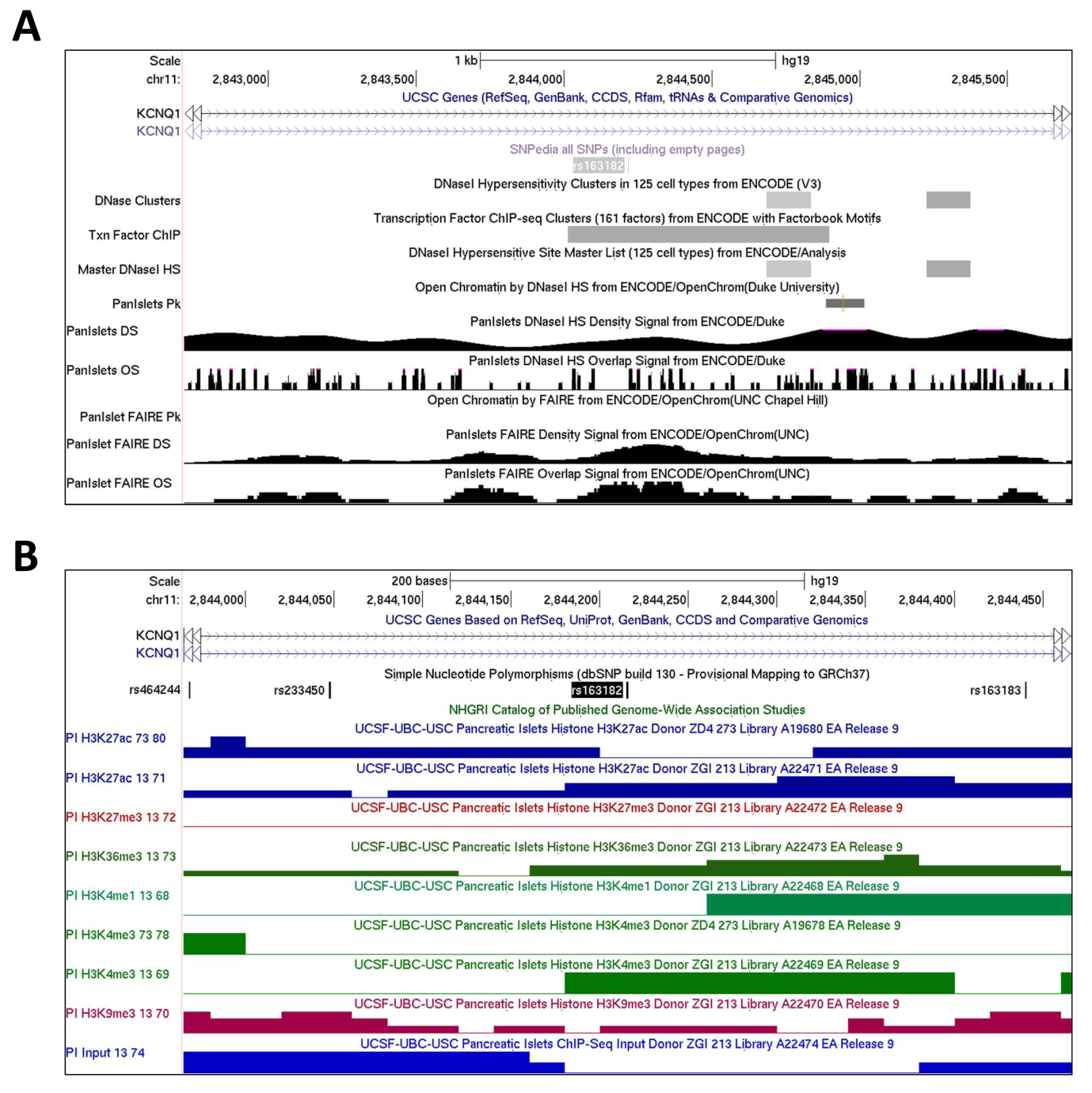


**Supplementary Figure 3. Chromatin features of rs163182.** Functional annotation in proximity to rs163182 in pancreatic islets from ENCODE (A) and Roadmap data (B). DNaseI hypersensitivity (HS) clusters, the Formaldehyde-Assisted Isolation of Regulatory Elements (FAIRE) density signal, and several histone modification markers (such as H3K27ac, H3K27me3, H3K36me3, H3K4me1, H3K4me3, and H3K9me3) are present.

**
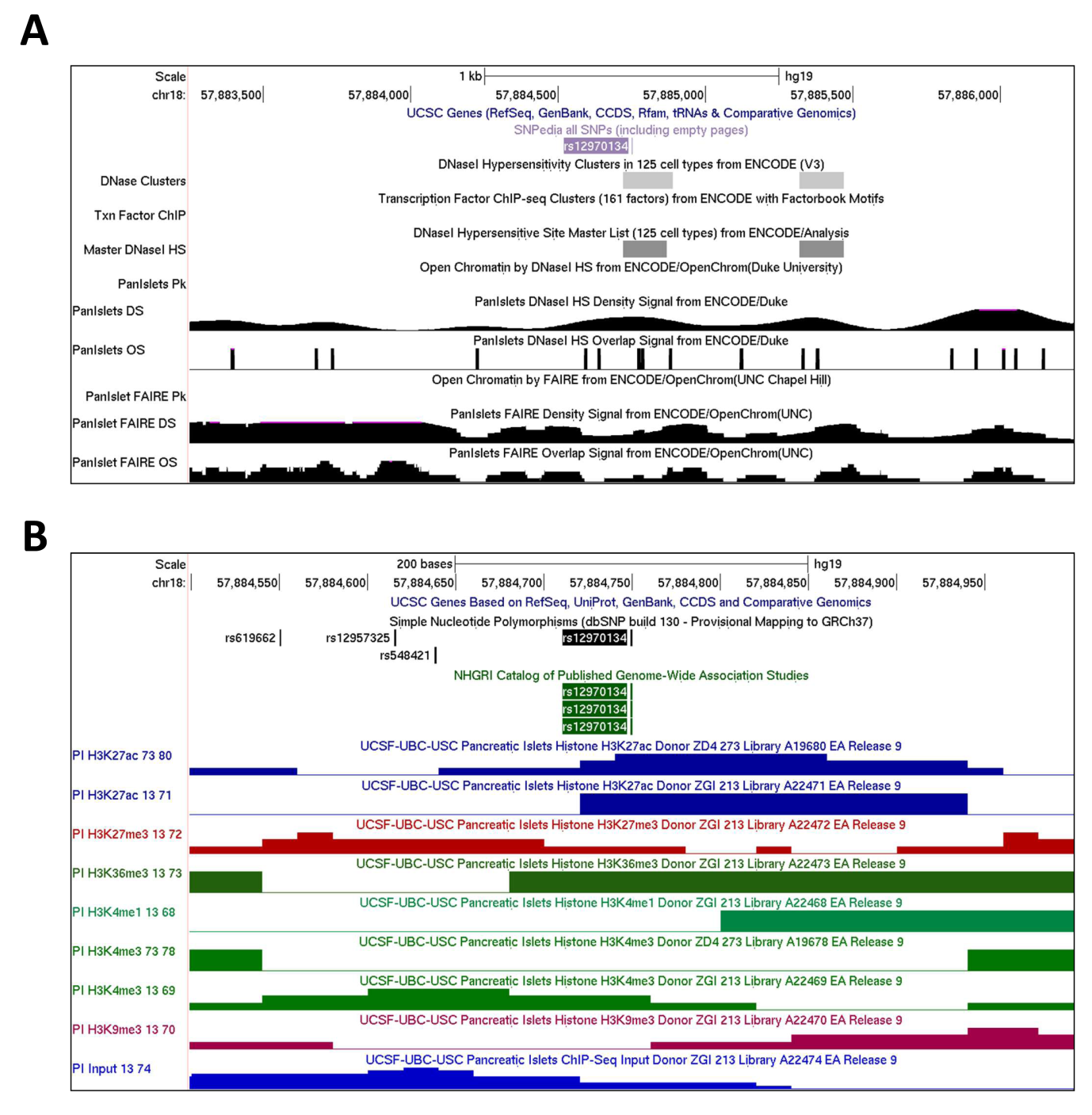
**

**Supplementary Figure 4. Chromatin features of rs12970134.** Functional annotation in proximity to rs12970134 in pancreatic islets from ENCODE (A) and Roadmap data (B). DNaseI hypersensitivity (HS) clusters, the Formaldehyde-Assisted Isolation of Regulatory Elements (FAIRE) density signal, and several histone modification markers (such as H3K27ac, H3K27me3, H3K36me3, H3K4me1, H3K4me3, and H3K9me3) are present.

**
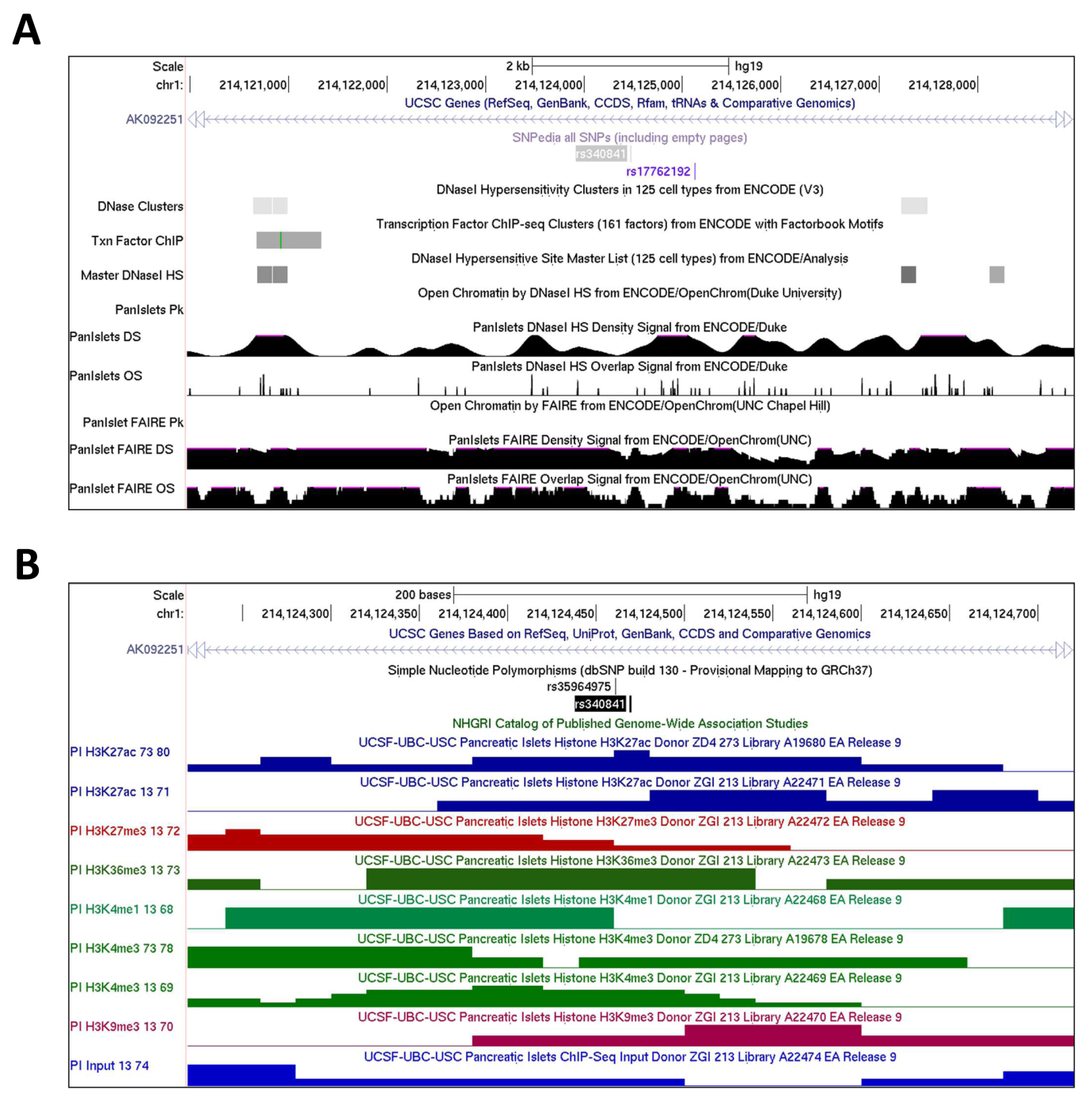
**

**Supplementary Figure 5. Chromatin features of rs340841.** Functional annotation in proximity to rs340841 in pancreatic islets from ENCODE (A) and Roadmap data (B). DNaseI hypersensitivity (HS) clusters, the Formaldehyde-Assisted Isolation of Regulatory Elements (FAIRE) density signal, and several histone modification markers (such as H3K27ac, H3K27me3, H3K36me3, H3K4me1, H3K4me3, and H3K9me3) are present.
